# Adipose tissue from metabolic syndrome mice induces an aberrant miRNA signature highly relevant in prostate cancer development

**DOI:** 10.1002/1878-0261.12788

**Published:** 2020-09-25

**Authors:** Cintia Massillo, Rocío Belén Duca, Ezequiel Lacunza, Guillermo Nicolás Dalton, Paula Lucía Farré, Nicolás Taha, Flavia Piccioni, Georgina Daniela Scalise, Kevin Gardner, Adriana De Siervi

**Affiliations:** ^1^ Laboratorio de Oncología Molecular y Nuevos Blancos Terapéuticos Instituto de Biología y Medicina Experimental (IBYME) CONICET Buenos Aires Argentina; ^2^ Centro de Investigaciones Inmunológicas Básicas y Aplicadas (CINIBA) Facultad de Ciencias Médicas Universidad Nacional de La Plata Buenos Aires Argentina; ^3^ Department of Pathology and Cell Biology Columbia University Medical Center New York NY USA

**Keywords:** adipose tissue, metabolic syndrome, miRNA, prostate cancer

## Abstract

Prostate cancer (PCa) remains an important public health concern in Western countries. Metabolic syndrome (MeS) is a cluster of pathophysiological disorders with increasing prevalence in the general population that is a risk factor for PCa. Several studies have determined that a crosstalk between white adipose tissue (WAT) and solid tumors favors cancer aggressiveness. In this work, our main goal was to investigate the interaction between WAT and PCa cells through microRNAs (miRNAs), in MeS mice. We developed a MeS‐like disease model using C57BL/6J mice chronically fed with high‐fat diet (HFD) that were inoculated with TRAMP‐C1 PCa cells. A group of five miRNAs (mmu‐miR‐221‐3p, 27a‐3p, 34a‐5p, 138‐5p, and 146a‐5p) were increased in gonadal WAT (gWAT), tumors, and plasma of MeS mice compared to control animals. Three of these five miRNAs were detected in the media from gWAT and TRAMP‐C1 cell cocultures, and significantly increased in MeS context. More importantly, hsa‐miR‐221‐3p, 146a‐5p, and 27a‐3p were increased in bloodstream of PCa patients compared to healthy donors. Using miRNA microarrays, we found that 121 miRNAs were differentially released to the coculture media between HFD‐gWAT and tumor cells compared to control diet‐gWAT and tumor cells. Target genes for the 66 most deregulated miRNAs were involved in common pathways, mainly related to fatty acid metabolism, ER protein processing, amino acid degradation, PI3K AKT signaling, and PCa. Our findings show for the first time a signature of five miRNAs as important players involved in the interaction between WAT and PCa in MeS mice. Further research will be necessary to track these miRNAs in the interaction between these tissues as well as their role in PCa patients with MeS.

## Graphical abstract

MicroRNAs (miRNAs) released by white adipose tissue (WAT) from metabolic syndrome (MeS)‐like disease mice impact on prostate cancer (PCa) growth. We identified a signature of five miRNAs (mmu‐miR‐221‐3p, 27a‐3p, 34a‐5p, 138‐5p, and 146a‐5p) as key players implicated in the interaction between WAT and PCa in MeS mice. Besides, these miRNAs were deregulated in tumors and bloodstream of PCa patients.

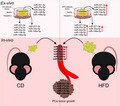

**Abbreviations**
BPH: benign prostatic hyperplasia
CD: control diet
CTBP1: C‐terminal binding protein 1
gWAT: gonadal white adipose tissue
HD: healthy donors
HFD: high‐fat diet
MeS: metabolic syndrome
miRNA: microRNA
NAT: normal adjacent tissue
PCa: prostate cancer
WAT: white adipose tissue

## 1 Introduction

Prostate cancer (PCa) is still the most prevalent cancer among men and the fifth cause of death by cancer worldwide [1]. Metabolic syndrome (MeS) is a disease whose diagnosis includes alteration of at least three of the following factors: visceral adiposity, triglycerides, high‐density lipoprotein levels, hypertension, and fasting glucose levels [2]. Several epidemiological studies have determined a significant correlation associating MeS with more aggressive PCa tumors and recurrence [3]. Nonetheless, the molecular mechanism responsible for the effect of MeS on PCa development is yet to be fully determined.

Our group identified C‐terminal binding protein 1 (CTBP1), a transcriptional corepressor, as a molecular link associating PCa and MeS. We previously generated a PCa and MeS‐like disease model by chronically feeding mice with a high‐fat diet (HFD). Thus, we identified novel pathways regulated by CTBP1 on a MeS environment [4, 5]. CTBP1 depletion in androgen‐insensitive PCa xenografts from HFD‐fed mice affects the expression of genes and microRNAs (miRNAs) involved in hormone biosynthesis, olfactory signaling, and cell adhesion pathways, impacting in PCa development and progression [6, 7, 8]. Additionally, an androgen‐sensitive PCa and MeS‐like mice model allowed us to determine that MeS significantly increased tumor growth. Compared to control diet (CD), HFD‐fed mice showed high amount of white adipose tissue (WAT), adipocyte size, and macrophage infiltration, with adipogenesis and inflammation‐related genes expression induction, which indicates chronic WAT inflammation, an important feature of MeS. Additionally, cocultures of androgen‐sensitive PCa cells with WAT determined that WAT from HFD‐fed mice induced proliferation and expression of oncogenes in PCa cells [7].

miRNAs are small noncoding RNAs (18–22 nts long) that target mRNA to reduce protein expression [9]. It was reported that miRNAs are important for WAT function regulating adipogenesis, metabolism, and signaling pathways [10], and probably released by WAT [10, 11]. Numerous studies have reported the role of miRNAs in cancer, including PCa [12]. It was reported that tumors release miRNAs into the bloodstream at different stages of the disease [12].

Several studies have determined that a crosstalk between WAT and solid tumors favors cancer aggressiveness. Mature adipocytes provide adipokines, hormones, and lipids to cancer cells, while stromal and immune cells from WAT locally secrete paracrine factors within the tumor microenvironment [13]. Moreover, it was suggested that in breast cancer, miRNAs from adipocytes gave rise to a niche from which, under aberrant conditions, a neoplastic transformation of breast cells may start [14]. Few studies investigated the interaction between WAT and PCa cells in a MeS microenvironment [15, 16]. In this work, our main goal was to explore the interaction between WAT and PCa cells through miRNAs in MeS mice.

## 2 Materials and methods

### 2.1 Cell culture and treatments

TRAMP‐C1 cell line (ATCC: CRL‐2730, Manassas, VA, USA) was grown in DMEM medium (GIBCO, Thermo Scientific, Beverly, MA, USA) supplemented with 10% of FBS, antibiotics, and 250 IU·μL^−1^ of human recombinant insulin.

### 2.2 Immunocompetent PCa allografts and MeS murine model

Four‐week‐old C57BL/6J male mice (*N* = 16) were housed under pathogen‐free conditions following the IBYME's animal care guidelines. Mice were randomized into two dietary groups and fed *ad libitum* during 27 weeks with CD (3120 kcal·kg^−1^, 5% fat) or HFD (4520 kcal·kg^−1^, 37% fat). After 15 weeks of diet, mice were injected s.c. with TRAMP‐C1 (3 × 10^6^) cell line. Animals were sacrificed in the 27th week, and tumor, WAT, and blood samples were collected. The metabolic state of the animals and tumor volume was previously reported [7].

### 2.3 *Ex vivo* coculture of gWAT with TRAMP‐C1 cells

TRAMP‐C1 cells were cocultured in 24‐Transwell plates with gWAT obtained from CD or HFD mice as previously described [7]. Briefly, TRAMP‐C1 cells were seeded in DMEM complete medium at a density of 1.5 × 10^4^ cells/well. After 24 h, medium was refreshed and 150 mg of gWAT obtained from CD or HFD C57BL/6J mice was placed onto cell‐culture inserts (3.0 μm pore size, high‐density membrane) in M199 (Sigma‐Aldrich, Darmstadt, Germany) complete medium for 48 h. Then, TRAMP‐C1 cells and gWAT were collected in TriReagent (Molecular Research Center, Cincinnati, OH, USA) for RNA isolation and RT–qPCR. Culture medium was collected for RNA isolation followed by miRNA microarray hybridization or RT–qPCR. As a control, TRAMP‐C1 cells were cultured without the addition of gWAT.

### 2.4 RNA isolation and reverse transcription (RT)

Total RNA from cells, allografts, WAT, or plasma was isolated using TriReagent (Molecular Research Center, Cincinnati, OH, USA). For plasma samples, cel‐miR‐39 synthetic miRNA (20–40 fmol) was spiked in before RNA isolation. miRNAs were retrotranscribed using the stem‐loop method as previously described [8, 17]. For RT, 100 ng (cells, allografts or WAT) or 4 μL (plasma o culture medium) of total RNA and 0.07 μm of stem‐loop primer were preheated (70 °C, 5 min). RT was performed using M‐MLV reverse transcriptase (Promega, Madison, WI, USA) and incubated in MyGenie96 Thermal Block (Bioneer, Daedeok‐gu, Daejeon, Republic of Korea) (30 min 16 °C, 50 min 37 °C, 15 min 70 °C).

### 2.5 Primer design for stem‐loop RT–qPCR

Primer design for stem‐loop RT–qPCR was performed following the guidelines described in Chen *et al*. work [17] and summarized at Table S1. Briefly, miRNA mature sequence was downloaded from miRBase database (http://www.mirbase.org/). The stem‐loop primer for RT was designed using a stem‐loop sequence (GTCTCCTCTGGTGCAGGGTCCGAGGTATTCGCACCAGAGGAGAC) followed by the last six nucleotides from the 5′ end of the mature miRNA. Forward primer for qPCR was designed using the mature sequence of the miRNA without the last six bases of the 5′ end. To extend the sequence, we added a short sequence formed by C and G to the 5′ end (e.g., CGCGCG). Likewise, the universal reverse primer was designed to match to the stem‐loop sequence. Tm, self‐complementarity, and off target products were assessed using the primer blast tool (https://www.ncbi.nlm.nih.gov/tools/primer‐blast/). As exclusion criteria, we chose primer pairs with an optimal Tm 60 °C with ±5 °C range and self‐complementarity and self‐3′ complementarity less than 4. For all primer pairs, the amplification efficiency was calculated and ranged between 90% and 105% which are adequate for relative quantification of expression by RT–qPCR using ΔΔ*C*_T_ method.

### 2.6 Real‐time PCR analysis

Tumors, cells and gWAT qPCRs were performed in 25 μL with 0.05–1 μL RT product, 1 U Taq DNA polymerase (Pegasus), 4 mm MgCl_2_, 0.2 mm dNTPs, 3 × 10^−5^ μL SYBR Green (Sigma‐Aldrich), 0.1 μm forward primer, and 0.1 μm reverse primer. The reactions were incubated in StepOne Plus Real Time PCR (Applied Biosystems, Beverly, MA, USA) (94 °C 2 min, 40 cycles: 95 °C 15 s, annealing temperature 20 s, 72 °C 25 s and 95 °C 15 s). Plasma and media qPCRs were run in 10 μL with 0.1 μm of each primer and 5 μL of PowerUp™ SYBR™ Green Master Mix (Thermo Fisher, Beverly, MA, USA), in StepOne Plus Real Time PCR (Applied Biosystems, Beverly, MA, USA) (50 °C 2min, 95 °C 10 min, 40 cycles: 95 °C 15 s, annealing temperature 15 s, 60 °C 1 min, and 95 °C 15 s). The expression levels of miRNAs were calculated using ΔΔ*C*_T_ method normalizing to geometric mean of mmu‐miR‐103a‐3p and 191‐5p and control (for tumor, cells, and gWAT). The expression levels of plasma were normalized to cel‐miR‐39 (a synthetic spike in added to the sample prior to RNA extraction). For culture media, we used mmu‐miR‐19b as a normalizer since it was detected with high signal in the microarray analyses and showed no changes between the experimental groups. Primer sequences are listed in Table S1.

### 2.7 Microarray analysis

RNA from coculture medium between TRAMP‐C1 cells and gWAT from CD and HFD‐fed mice was isolated with NucleoSpin^®^ miRNA Plasma (Macherey‐Nagel, Düren, Germany) kit and hybridized with GeneChip^®^ miRNA 4.0 Array (Affymetrix, Santa Clara, CA, USA) (*N* = 2 per group). For miRNA expression analysis, we employed the Limma and pd.mirna.4.0 packages in the r/bioconductor (Roswell Park Comprehensive Cancer Center, Buffalo, NY, USA) environment. Firstly, CEL files were uploaded into r; to read and normalize the data, we used the gcrma package, while for annotation, we used the pd.mirna.4.0 package. The gcrma function adjusts for background intensities which include optical noise and nonspecific binding. gcrma converts background adjusted probe intensities to expression measures using the same normalization and summarization methods as rma (Robust Multiarray Average), in which raw intensity values are background corrected, log2 transformed, and then quantile normalized. For differential expression analysis, we used the rank product method for two‐class unpaired data and an alpha critical *P* value of 0.01 [18]. For miRNA pathway analysis, a list of experimentally validated target genes miRNA, derived from diana‐tarbase v7 (DIANA LAB, University of Thessaly, Filellinon, Volos, Greece), was obtained with the diana‐mirpath v3tool (DIANA LAB, University of Thessaly) for DE miRNAs (LogFC > 1, *P* value > 0.01). Functional enrichment analysis of target genes was performed with the ClueGo plug‐in of the cytoscape software [19] in order to capture the biological processes associated with DE miRNAs.

### 2.8 TCGA dataset analysis

MiRNA expression of prostate tumors of patients was obtained from the TCGA Prostate Cancer (PRAD) cohort using ucsc xena resource (http://xena.ucsc.edu/) [20]. Fifty‐two PCa samples and 52 normal adjacent tissues (NAT) were included in the present study excluding patients without this information. Clinical‐pathological (e.g., age, risk category) and survival data (overall and event‐free survival) for these patients were obtained using the r package ggpubr (clinical‐pathological data of patients are in Table S2). For miRNA mature strand expression, the miRNA‐Seq (IlluminaHiSeq_miRNASeq) data were downloaded as log2 (RPM + 1) values. For statistical analysis, normality of data was assessed using Shapiro–Wilk test and homogeneity of variances was analyzed by boxplot. If data fulfill the requirements, a paired sample t‐test was used. Otherwise, Sign Median test was performed using singmedian.test r package. The results shown here are in whole based upon data generated by the TCGA Research Network: https://www.cancer.gov/tcga.

### 2.9 Circulating miRNA profile analysis

Circulating miRNA expression profile was analyzed in the plasma of a cohort of 60 PCa patients and 78 controls [51 men with benign prostatic hyperplasia (BPH) or 27 healthy donors (HD)] using microarray data available from Mello‐Grand *et al*. [21]. Average normalized log2Intensities data (GSE113234) was downloaded from the Gene Expression Omnibus (GEO) public functional genomic data repository (https://www.ncbi.nlm.nih.gov/geo/) and analyzed using the ggpubr r package. To compare data between PCa and non‐PCa, one‐way analysis of variance (one‐way ANOVA) with Tukey's multiple comparisons test was applied. GSE113234 was generated by Agilent Technologies‐Human miR Microarray version 19 (Agilent Technologies, Santa Clara, CA, USA).

### 2.10 Statistical analysis

For the *in vivo* (*N* = 8 per group) and the *ex vivo* experiments (*N* = 3 per group), the results are given as mean and standard deviation (SD) or standard error of the mean (SEM). For statistical analysis, normality and homogeneity of variances were assessed using Shapiro–Wilk and Levene tests, respectively. If data fulfill the requirements, Student's *t*‐test or one‐way ANOVA followed by Tukey was used. Significance level of 5%.

## 3 Results

### 3.1 MeS modulates miRNA expression in gWAT from mice

**Fig. 1:**
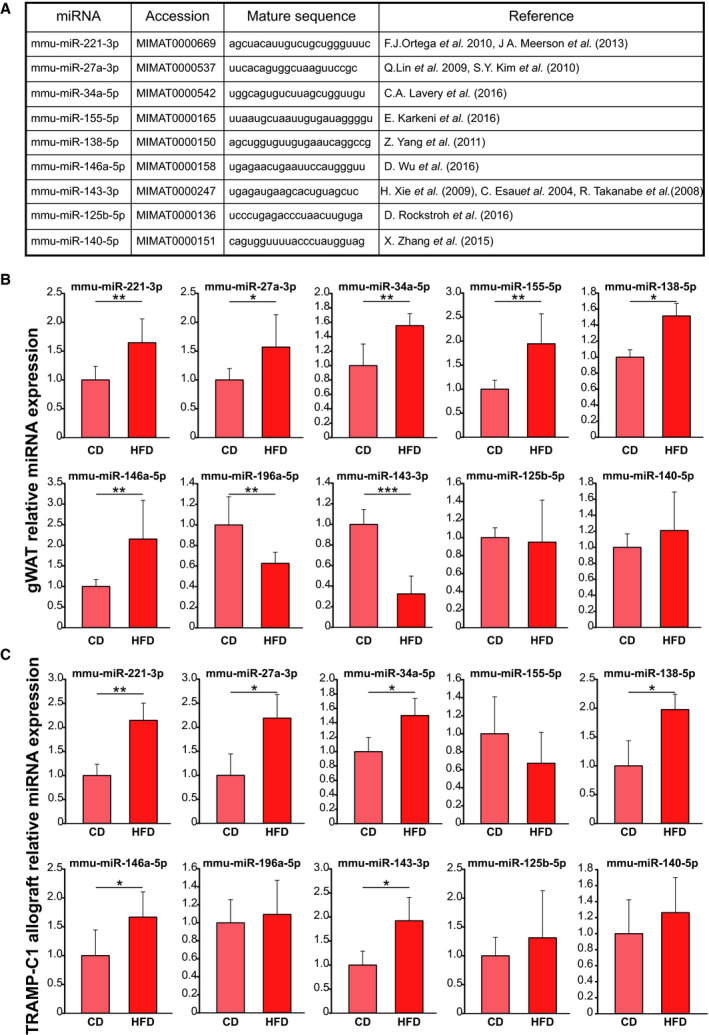
MeS modifies the expression of miRNAs in gWAT and androgen‐sensitive prostate tumors from mice. (A) Table showing a list of miRNAs involved in adipogenesis regulation and adipocyte metabolism selected from literature. Stem‐loop RT–qPCR from gWAT (B) and TRAMP‐C1 allografts (C) obtained from CD‐ or HFD‐fed C57BL/6J mice using specific primers for the indicated miRNAs is shown (*N* = 8 per group). Data were normalized to geometric mean of miR‐191‐5p and miR‐103a‐3p and control and expressed as mean and SD. Statistical analysis was performed using two‐sided *t*‐test. *P < 0.05; **P < 0.01; ***P < 0.001.

Previously, we reported that MeS significantly increased TRAMP‐C1 tumor growth and oncogene expression in C57BL/6J mice chronically fed with HFD. Additionally, MeS mice showed altered morphology and increased amount of WAT, with adipogenesis/inflammation gene expression induction [7]. Here, we investigated the effect of MeS on miRNA expression profile from gWAT. Based on the literature, we selected 10 miRNAs involved in adipogenesis regulation and adipocyte metabolism that were reported as deregulated in obesity or involved in adipocyte differentiation detailed in Fig. 1A. We measured the expression of these miRNAs in the gWAT derived from HFD and CD mice. Results showed significantly increase in the expression of mmu‐miR‐221‐3p, 27a‐3p, 34a‐5p, 155‐5p, 138‐5p, and 146a‐5p, and decrease in mmu‐miR‐196a‐5p and 143‐3p levels in HFD‐gWAT compared to CD mice (Fig. 1B). No changes were detected in mmu‐miR‐125b‐5p and 140‐5p expression levels (Fig. 1B).

### 3.2 MeS induces oncomiR expression in androgen‐sensitive murine prostate tumors

We determined the expression levels of the selected miRNAs in TRAMP‐C1 tumors developed in HFD‐ and CD‐fed mice. Most of the miRNAs altered in gWAT were induced in TRAMP‐C1 allografts from HFD‐fed mice (mmu‐miR‐221‐3p, 27a‐3p, 34a‐5p, 138‐5p, 146a‐5p, and 143‐3p) compared to CD‐fed mice (Fig. 1C). No significant changes were observed in mmu‐miR‐155‐5p, 196a‐5p, 125b‐5p, and 140‐5p expression between diet groups.

### 3.3 MeS increases mmu‐miR‐221‐3p, 27a‐3p, 34a‐5p, 138‐5p, and 146a‐5p in mice bloodstream

**Fig. 2:**
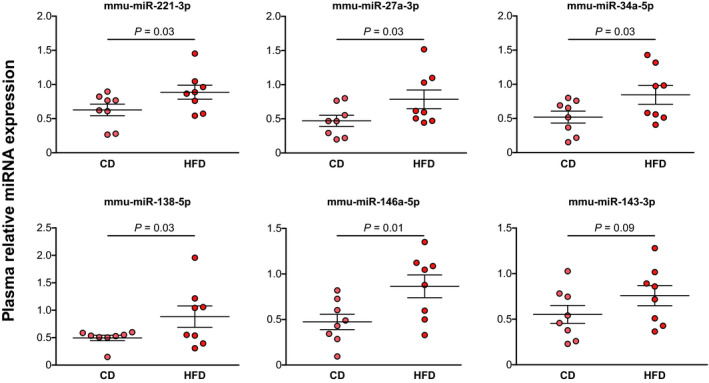
MeS increases miRNA circulation in plasma from mice. Mouse plasma samples were analyzed by stem‐loop RT–qPCR for the indicated miRNAs and normalized to spike in cel‐39 synthetic miRNA (*N* = 8 per group). $2^{‐\Delta C_{t}}$ is graphed ($\Delta C_{t}\;\text{value}=‐C_{t_{\text{sample}}}‐C_{t_{\text{cel}‐\text{39}}}$) and expressed as mean and SEM. Data were analyzed by one‐sided *t*‐test.

miRNAs are secreted as stabilized factors into the bloodstream, as circulating miRNAs, and function as endocrine messengers given their release and uptake by recipient tissues throughout the body [11]. We hypothesized that miRNA expression increased in tumors might be due to the release of miRNAs from gWAT into bloodstream, which might be uptaken by distant PCa cells. Therefore, we isolated the total miRNAs from HFD and CD mice plasma and measured those that were altered in gWAT and tumors. We found that five miRNAs: mmu‐miR‐221‐3p, 27a‐3p, 34a‐5p, 146a‐5p, and 138‐5p were increased in plasma from HFD‐fed mice compared to control animals (Fig. 2). These findings indicate that these miRNAs not only circulate in mice blood but also have a distinctive circulation level in a MeS context.

### 3.4 gWAT from MeS mice releases miRNAs that might impact on PCa growth

**Fig. 3:**
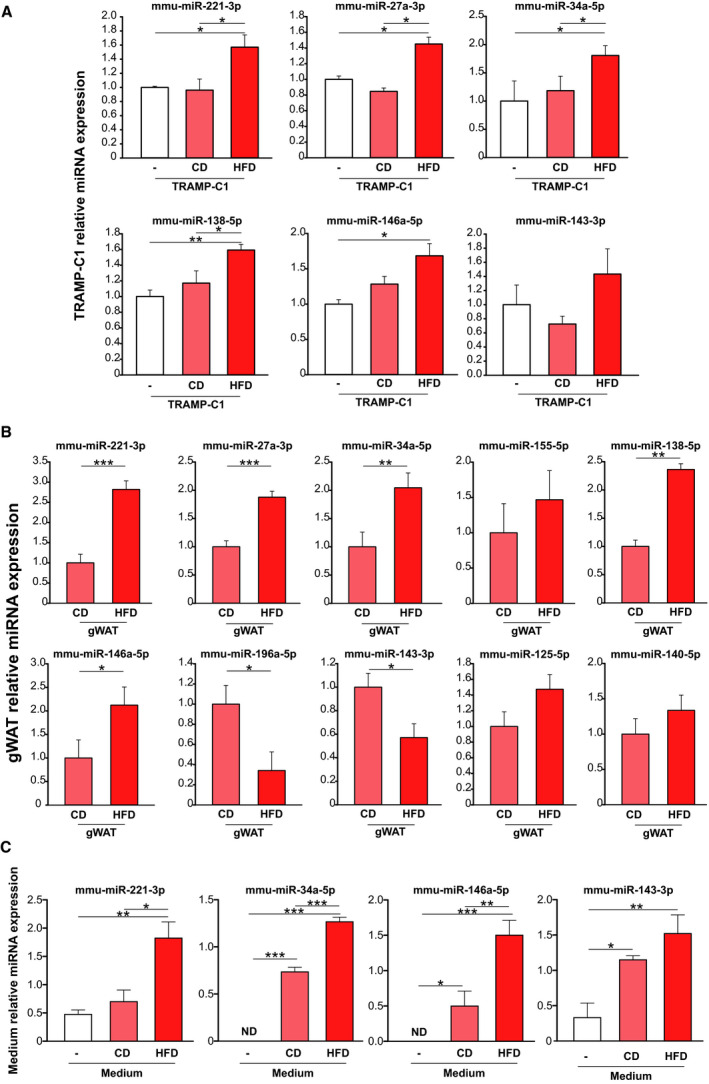
gWAT from MeS mice releases miRNAs impacting in prostate tumor growth. (A) Stem‐loop RT–qPCR from TRAMP‐C1 cells obtained from the coculture using specific primers for the indicated miRNAs. Data were normalized to geometric mean of miR‐191‐5p and miR‐103a‐3p and control (TRAMP‐C1 cells without the addition of gWAT) and represent mean and SD of three independent experiments with three replicates. Data were analyzed by one‐way ANOVA followed by Tukey's. (B) Stem‐loop RT–qPCR from gWAT obtained from the coculture. Data were normalized to geometric mean of miR‐191‐5p and miR‐103a‐3p and to CD group. Data were analyzed by two‐sided *t*‐test. (C) Stem‐loop RT–qPCR in culture medium from cocultures using primers specific for the indicated miRNAs. $2^{‐\Delta C_{t}}$ is graphed. Data were normalized to mmu‐miR‐19b ($\Delta C_{t}\;\text{value}=‐C_{t_{\text{sample}}}‐C_{t_{\text{miR-}‐\text{19b}}}$) and control (TRAMP‐C1 cells without the addition of gWAT) and represent the mean and SD of three independent experiments with three replicates. Data were analyzed by one‐way ANOVA followed by Tukey's. ND: not detectable. *P < 0.05; **P < 0.01; ***P < 0.001. White bar corresponds to the group of TRAMP‐C1 without the addition of gWAT, pink bar corresponds to the TRAMP‐C1 cell culture with gWAT from CD mice, and red bar corresponds to TRAMP‐C1 cultured with gWAT from HFD mice.

We previously reported that gWAT from HFD‐fed mice cocultured with TRAMP‐C1 cells induced proliferation and oncogene expression compared to CD‐gWAT [7]. Here, we performed an *ex vivo* coculture assay, in which TRAMP‐C1 cells were cocultured with gWAT from CD‐ or HFD‐fed mice. TRAMP‐C1 cells cultured without gWAT were used as control. After 48 h, TRAMP‐C1 cells, gWAT, and coculture media were harvested and RT–qPCR was performed. Interestingly, we found that gWAT from HFD‐fed mice increased the levels of mmu‐miR‐221‐3p, 27a‐3p, 34a‐5p, 138‐5p, and 146a‐5p in TRAMP‐C1 cells (Fig. 3A), reproducing the same effect observed in the allografts from HFD‐fed mice (Fig. 1B). Likewise, gWAT from HFD‐fed mice showed mmu‐miR‐221‐3p, 27a‐3p, 34a‐5p, 138‐5p, and 146a‐5p expression induction, mmu‐miR‐196a‐5p and 143‐3p repression levels and no changes for mmu‐miR‐155‐5p and 125b‐5p compared to CD mice (Fig. 3B). Moreover, mmu‐miR‐221‐3p, 34a‐5p, and 146a‐5p were released to the coculture medium and significantly increased in the interaction between HFD‐gWAT/TRAMP‐C1 cells compared to CD‐gWAT/TRAMP‐C1 cells (Fig. 3C). Altogether, these findings support a novel crosstalk between WAT and PCa cells, through miRNA release, in MeS mice.

### 3.5 Crosstalk between gWAT and PCa cells by miRNA release from MeS mice

Results described above would indicate a differential release of miRNAs, depending on whether tumor cells are cocultured with CD of HFD‐gWAT. To further understand the differential release of miRNAs, we conducted a miRNA microarray analysis with miRNAs isolated from the coculture medium between TRAMP‐C1 cells with HFD‐gWAT *versus* TRAMP‐C1 cells with CD‐gWAT.

**Fig. 4:**
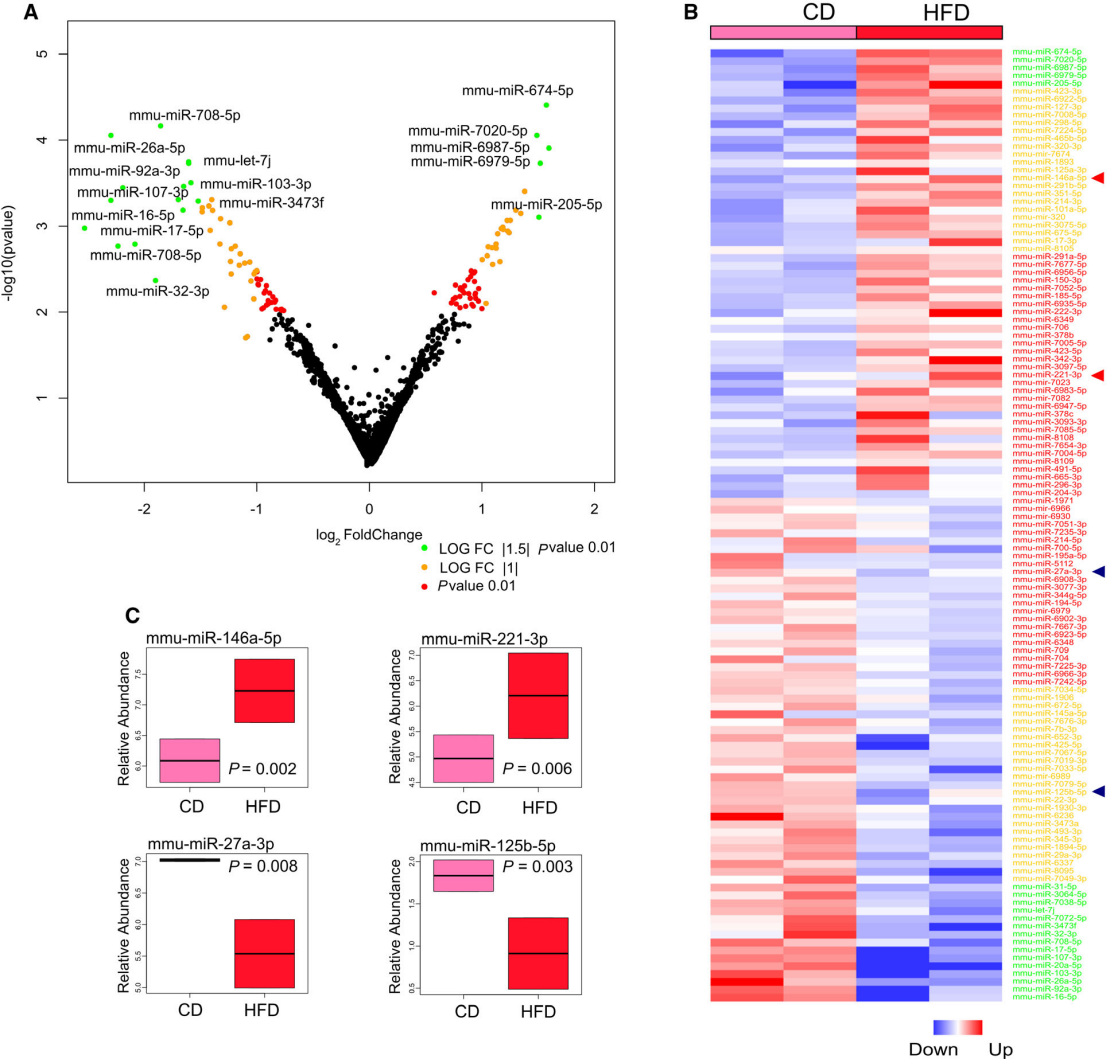
Crosstalk between gWAT and prostate tumor cells by miRNAs released in a MeS environment. (A) Volcano plot representation of DE miRNAs. Colors indicate the levels of significance. The names of some of the most significant miRNAs are indicated. (B) Heat map representation of DE miRNAs. Arrows indicate the four miRNAs common to the 10 selected miRNAs based on the literature. (C) Box plots of the expression of the four selected miRNAs.

The analysis showed a total of 121 miRNAs identified as differentially abundant. Fifty‐seven of these miRNAs showed an increase in coculture medium of HFD compared with CD group, while 64 showed a decrease (Fig. 4, Table S3). As shown in the volcano plot, three groups of miRNAs based on statistical significance *versus* magnitude of change were established: Log FC > 1.5, *P* value < 0.01 (green, *n* = 20); LogFC > 1, *P* value < 0.01 (yellow, *n* = 46); and *P* value < 0.01 (red, *n* = 55) (Fig. 4A, Table S3). We searched whether any of the 10 miRNAs selected according to the literature (Fig. 1A) were among the deregulated ones. Interestingly, we found four of them, mmu‐miR‐221‐3p and 146a‐5p, among the upregulated in HFD, in the groups yellow and red, respectively; and mmu‐miR‐27a‐3p and 125b‐5p in the groups yellow and red of the downregulated, respectively (Fig. 4B,C), which supports the relevance of these miRNAs in the interaction between WAT and PCa cells in a MeS environment.

**Fig. 5:**
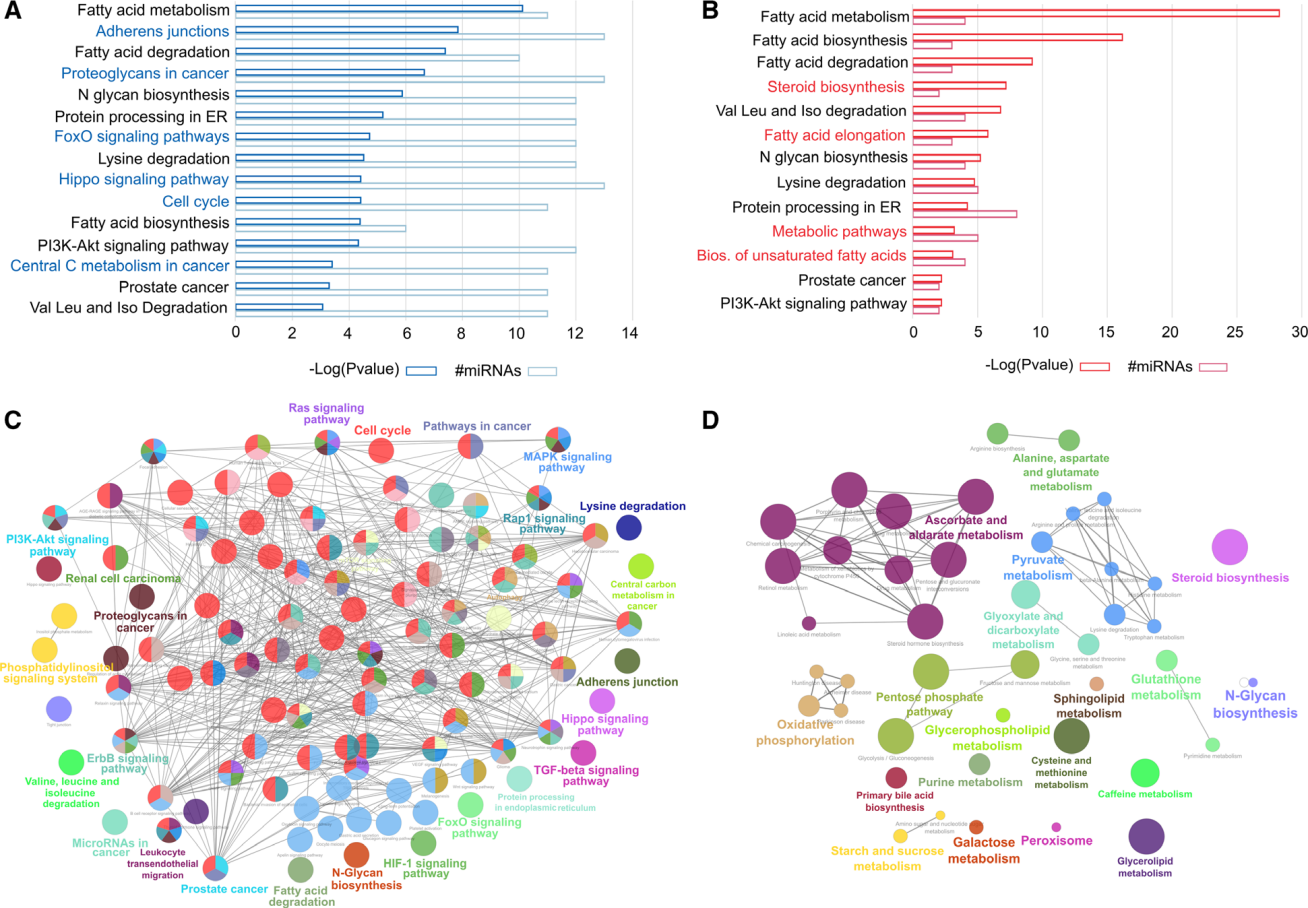
Functional enrichment analysis of validated miRNA targets. (A, B) Barplot representation of the top significant KEGG pathways associated with the target genes of the downmodulated (A) and upmodulated (B) miRNAs, released to the medium of the coculture of TRAMP‐C1 cells with gWAT from HFD‐fed mice compared to CD‐fed mice. The terms common to both groups are indicated in black, and those specific to each group are highlighted in color. (C, D) Functional enrichment analysis represented as nonredundant biological terms in a functionally grouped network of miRNA targets exclusive of the downmodulated (C) and upmodulated miRNAs (D).

To explore the functional role of the differentially abundant miRNAs and identify controlled pathways, we used diana‐mirpath v3 tool. Since each miRNA can control up to dozens of genes, we selected only the most deregulated miRNAs—the green and orange zones (*n* = 66; Fig. 4B)—to obtain a more robust result. In addition, we employed diana‐tarbase v7 of the miRPath tool, which is a reference database of experimentally supported miRNA targets. Seventeen of the downmodulated (17/40) and 13 of the upmodulated miRNAs (13/26) showed validated target genes (Table S4). For each group of genes, KEGG signaling pathways were identified with a *P* value < 0.01 (Fig. 5, Tables S5 and S6). Target genes of both down‐ and upmodulated miRNAs shared common KEGG pathways, mainly related to fatty acid metabolism, ER protein processing, amino acids degradation, PI3K AKT signaling, and PCa (Fig. 5A,B). Both lists of genes also showed their particular pathways: Those derived from downmodulated miRNAs enriched processes associated with Cell cycle, FoXO signaling, Hippo signaling, and other cancer‐related pathways (Fig. 5A), while those derived from up‐miRNAs were more associated with processes related to lipid and steroid biosynthesis and metabolic pathways (Fig. 5B). Redundancy obtained means that different miRNAs can have the same target, or different targets can belong to the same pathway. Therefore, we subtracted the common genes to both lists and performed a new functional enrichment (Tables [Link], [Link], [Link]). Interestingly, we observed that targets of downmodulated miRNAs were preferentially associated with cancer processes and related pathways (*P* < 0.001; Fig. 5C), whereas upmodulated miRNAs showed enrichment in specific metabolic pathways (*P* < 0.001; Fig. 5D).

Overall, miRNAs obtained from the coculture medium modulate a large repertoire of genes that affect the functionality of specific metabolic and cancer‐related pathways.

### 3.6 hsa‐miR‐221‐3p, 27a‐3p, 146a‐5p, and 140‐5p are increased in bloodstream of PCa patients

**Fig. 6:**
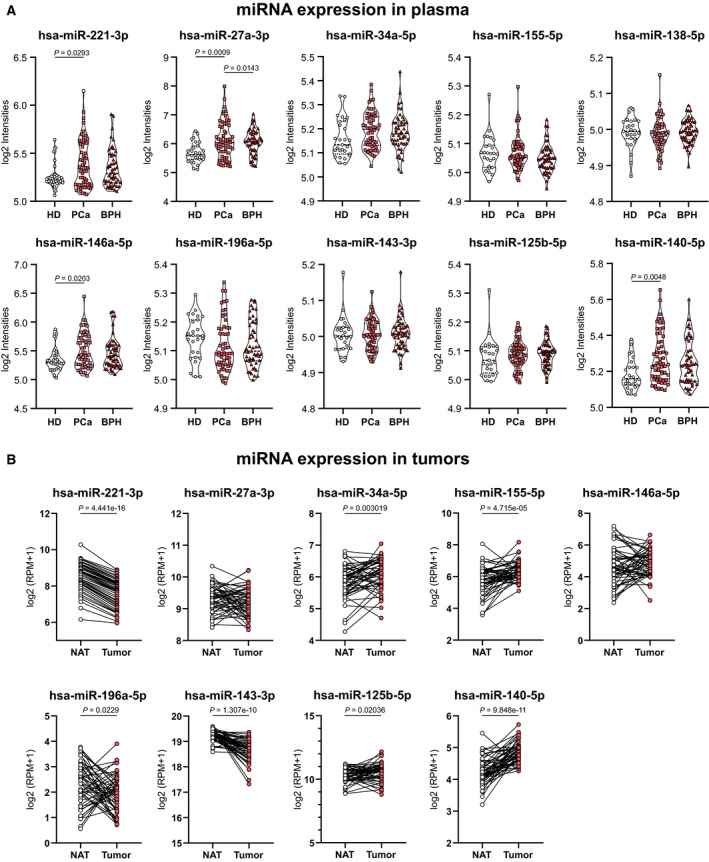
Differential miRNA expression in tumor tissue and circulation of PCa patients. (A) Circulating miRNA expression levels of hsa‐miR‐221‐3p, 27a‐3p, 34a‐5p, 155‐5p, 138‐5p, 146a‐5p, 196a‐5p, 143‐3p, 125b‐5p, and 140‐5p in plasma of PCa and non‐PCa patient samples. Log2Intensities values are plotted. Data were analyzed by one‐way ANOVA. (B) Expression levels of hsa‐miR‐221‐3p, 27a‐3p, 34a‐5p, 155‐5p, 146a‐5p, 196aa‐5p, 143‐3p, 125b‐5p, and 140‐5p in prostate primary solid tumor and NAT. Read per millions values are graphed. Data were analyzed using paired *t*‐test.

Since the discovery of miRNAs, many research groups have analyzed cancer patient blood samples in hopes of establishing novel biomarkers of this disease. In this sense, Mello‐Grand group performed a miRNA expression profile in 130 PCa patients, benign prostate hyperplasia (BPH) and HD blood samples using microarray technologies. As most of the miRNAs from mouse can be found in humans with similar function, in this work, we used the microarray results available from Mello‐Grand work to explore the expression of the selected miRNAs from Fig. 1 in plasma samples of a cohort of 60 PCa and 70 HD + BPH patients. Our analysis evidenced that hsa‐miR‐221‐3p, 146a‐5p, and 140‐5p were increased in bloodstream of PCa patients compared to HD but not with BPH subjects (Fig. 6A). Moreover, hsa‐miR‐27a‐3p was upregulated in PCa samples compared to HD and BPH samples (Fig. 6A), suggesting that this miRNA would be useful to distinguish not only PCa patients from HD, but also PCa patients from BPH individuals.

### 3.7 miRNAs deregulated in prostate tumors from patients

In order to further understand the pattern of expression of miRNAs between plasma and tumors, we performed a bioinformatical analysis using ucsc xena resource to determine the expression of the selected miRNAs (Fig. 1A) in prostate tumors respect to NAT. As shown in Fig. 6B, we found hsa‐miR‐34a‐5p, 155‐5p, 125b‐5p, and 140‐5p significantly overexpressed and hsa‐miR‐221‐3p, 196a‐5p, and 143‐3p significantly downregulated in PCa compared to NAT (Fig. 6B). Moreover, we did not find expression data for hsa‐miR‐138‐5p, and hsa‐miR‐27a‐3p and 146a‐5p showed no changes when we compared PCa tissue and NAT.

## 4 Discussion

**Fig. 7:**
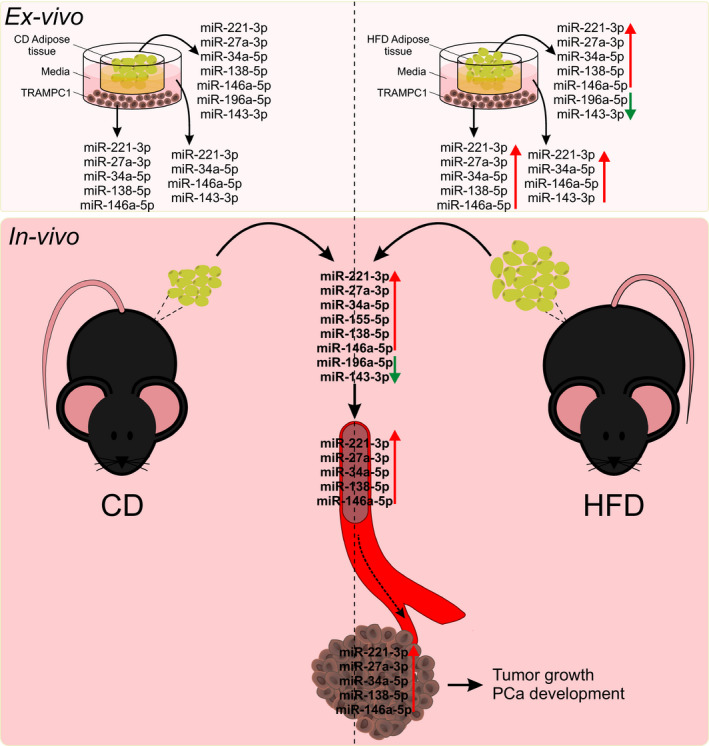
Hypothetical model. Upper panel: *ex vivo* cocultures between TRAMP‐C1 cells and adipose tissue from CD‐ or HFD‐fed mice. miRNAs induced (red arrow) or decreased (green arrow) in the cells, gWAT, and media when compared CD *versus* HFD groups are listed. Lower panel: miRNAs up‐ (red arrow) or downregulated (green arrow) in the *postmortem* adipose tissue, plasma, and allografts from CD‐ or HFD‐fed mice.

In this work, we provide evidence of a striking crosstalk between WAT and androgen‐sensitive PCa, through miRNAs in MeS mice. We found that MeS modulates the expression of several miRNAs in prostate tumors and gWAT implicated in cancer development and progression. In particular, we found a group of 5 miRNAs (mmu‐miR‐221‐3p, 27a‐3p, 34a‐5p, 138‐5p, and 146a‐5p) that were increased in gWAT, tumors, and plasma of MeS mice compared to control animals (Fig. 7). Interestingly, three of these five miRNAs were also detected in the media from gWAT and TRAMP‐C1 cell cocultures, and significantly increased in MeS context (Fig. 7).

To better understand the scope of these findings, we analyzed the expression of the selected miRNAs (Fig. 1A) in human datasets. We found that three of the five miRNAs regulated in mice (hsa‐miR‐221‐3p, 146a‐5p, and 27a‐3p) were increased in blood of PCa patients compared to HD. Moreover, from these, only hsa‐miR‐221‐3p and 34a‐5p were identified as deregulated in PCa samples. These results indicate that at least miR‐221‐3p, 146a‐5p, and 27a‐3p, detected in mice, were also high in bloodstream of PCa patients. However, of these three miRNAs, only hsa‐miR‐221‐3p was found to be inversely deregulated in PCa compared to normal tissues, which demonstrate that the expression of miRNAs in tumors does not always correlate with their expression in bloodstream. Similarly, Zedan *et al*. [22] reported that miR‐221‐3p is downregulated in primary metastatic prostate cancer (mPCa) tissue and upregulated in plasma from mPCa patients compared to the control cohort. Although these results seem to be contradictory, it is important to highlight that neither the source nor the mechanism of miRNAs released into the extracellular environment or body fluids is completely understood. There are several hypotheses to explain the source of circulating miRNAs: passive release of free miRNAs, as a part of ribonucleoprotein complexes (bound to proteins of the Ago family) within apoptotic bodies or inside microvesicles or exosomes [23]. Also, it has been proposed that extracellular miRNAs can be generated from immune cells in the tumor microenvironment [24, 25]. In this context, it would be interesting to investigate whether the expression pattern of the proposed miRNAs in this study in PCa tumors correlates with miRNA expression in vesicles or Ago bound in plasma or serum.

Different studies showed the expression and function of mmu‐miR‐221‐3p, 27a‐3p, 34a‐5p, 138‐5p, and 146a‐5p in WAT in MeS environment: For instance, mmu‐miR‐221‐3p and 146a expression was increased in gWAT from HFD‐fed mice [26, 27, 28, 29, 30]; mmu‐miR‐146a controls inflammation and prevents obesity onset when mice are fed a HFD [31] and hsa‐miR‐221‐3p contributes insulin resistance development that typically correlates with obesity [28, 29]; hsa‐miR‐34a‐5p expression was increased during adipogenesis and associated with high body weight index [29]; and hsa‐miR‐138 expression is diminished during adipocyte differentiation [32].

The miRNA 221 is one of the most promising biomarkers for PCa diagnosis and prognosis. It is induced in PC3 cells and trigger clonogenicity of this cell line [33, 34] and *in vivo* growth of androgen‐sensitive LNCaP cells [34]. While experimental models establish to hsa‐miR‐221 as an oncomiR, studies on patient samples showed contradictory results. Similar to our findings, some studies indicate that hsa‐miR‐221‐3p is decreased in PCa in comparison with normal samples [35, 36, 37]. However, other reports showed that this miRNA is increased in PCa compared to normal tissues [34, 38]. Likewise, several reports propose to hsa‐miR‐221‐3p as a good biomarker for diagnosis, progression, and response to therapy in PCa [36, 39, 40, 41].

Thus, based on our and other groups' findings, we propose to hsa‐miR‐221‐3p as a promising miRNA to be study as a potential biomarker for PCa diagnosis and prognosis.

In addition to our work, a previous study of Nara *et al*. identified 38 up‐ and 21 downregulated miRNAs in an androgen‐sensitive PCa model (LNCaP xenografts) under HFD conditions using microRNA arrays. Specifically, miR‐130a was attenuated in HFD‐induced PCa progression and modulated MET expression in PCa cells [42]. Therefore, the alteration of miRNAs in prostate tumors in a HFD environment could favor PCa development and progression.

In this work, we conducted a miRNA microarray analysis with miRNAs obtained from the coculture medium between TRAMP‐C1 cells with HFD‐gWAT *versus* TRAMP‐C1 cells with CD‐gWAT. The analysis revealed 57 increased and 64 decreased miRNAs in coculture medium of HFD compared with CD group. When we compared the controlled pathways by the 66 most deregulated miRNAs differentially released, we found target genes of both, down‐ and upmodulated miRNAs, regulated pathways related to fatty acid metabolism, ER protein processing, amino acid degradation, PI3K/AKT signaling, and PCa. Therefore, miRNAs release to the coculture medium would modulate a large plethora of genes that regulates specific metabolic and cancer‐related pathways.

Assuming that miRNAs regulate gene expression by silencing RNA of their target genes, the decrease in miRNAs released into the circulating medium, whose targets are genes of the cell cycle, and/or pathways related to cancer, may favor the deregulation of these genes, promoting tumor growth.

It is worth to note the paradoxical expression pattern of some miRNAs between PCa cells, gWAT, and coculture medium. For instance, while mmu‐miR‐125‐5p levels do not differ in gWAT neither in TRAMP‐C1 cells from HFD compared to CD mice in cocultures, it was found to be downregulated in the coculture media of HFD‐gWAT/TRAMP‐C1 cells compared to CD‐gWAT/TRAMP‐C1 using microarrays. It is important to mention that we were not able to detect this miRNA by RT–qPCR in the coculture media; therefore, these results should be taken with caution. Differences in methods applied in cells, gWAT (PCR), and culture medium (microarrays) may explain in part this inconsistency. More studies are necessary to understand the scope of these findings and the source of all the released miRNAs.

In summary, our findings show for the first time a group of five miRNAs (mmu‐miR‐221‐3p, 27a‐3p, 34a‐5p, 138‐5p, and 146a‐5p) as important players involved in the interaction between WAT and PCa in MeS mice. From these, hsa‐miR‐221‐3p, 27a‐3p, 34a‐5p, and 146a‐5p were confirmed to be important in PCa patients. Further research will be necessary to track these miRNAs in the interaction between these tissues, as well as their role in PCa patients with MeS.

## 5 Conclusions

Our study describes for the first time a signature of 5 miRNAs (mmu‐miR‐221‐3p, 27a‐3p, 34a‐5p, 138‐5p, and 146a‐5p) as important players involved in the interaction between WAT and PCa in MeS mice. Moreover, four of these miRNAs were confirmed to be important in PCa patients.

## Conflict of interest

The authors declare no conflict of interest.

## Author contributions

CM and ADS designed the study. CM, RBD, GND, PLF, FP, and GDS carried out the experiments. EL and NT performed bioinformatic analyses. CM, RBD, EL, and ADS interpreted the data. CM and ADS wrote the manuscript with contributions of KG and approval from all authors.

## Supporting information


**Table S1.** Primer sequences.

**Table S2.** Clinical‐pathological data of patients.

**Table S3.** Deregulated miRNAs.

**Table S4.** miRNAs with LOGFC>1, pvalue<0.05 and their targets.

**Table S5.** Functional enrichment of the down modulated miRNAs.

**Table S6.** Functional enrichment of the up modulated miRNAs.

**Table S7.** Lists of target genes associated to deregulated miRNAs.

**Table S8.** Functional enrichment of target genes present only in down modulated miRNAs.

**Table S9.** Functional enrichment of target genes present only in up modulated miRNAs.

## Data Availability

Microarray data have been deposited in GEO database (accession number GSE149506).
