# Supplementary material for: Adipose tissue from metabolic syndrome mice induces an aberrant miRNA signature highly relevant in prostate cancer development
Source: Mol Oncol. 2020 Sep 25;14(11):2868–83. doi: 10.1002/1878-0261.12788 (PMC7607170; doi:10.1002/1878-0261.12788)
Supplement: Supplementary file 1 — Table S1. Primer sequences. [file MOL2-14-2868-s001.pdf]

Table S1. Primer sequences used for stem-loop RT-qPCR

Primer	Sequence (5’-3’)	T_{ann} (°C)
RT-Stem-loop-Rv	TGGTGCAGGGTCCGAGGTATT	--
RT-mmu-miR-196a-5p-STEM	GTCTCCTCTGGTGCAGGGTCCGAGGTATTCGCACCAG AGGAGACCCCAAC	--
RT-mmu-miR-196a-5p Fw	CGGGCGGTAGGTAGTTTCATGTT	65
RT-mmu-miR-146a-5p-STEM	GTCTCCTCTGGTGCAGGGTCCGAGGTATTCGCACCAG AGGAGACAACCCA	--
RT-mmu-miR-146a-5p Fw	CGGGCGGTGAGAACTGAATTCC	65
RT-mmu-miR-34a-5p-STEM	GTCTCCTCTGGTGCAGGGTCCGAGGTATTCGCACCAG AGGAGACACAACC	--
RT-mmu-miR-34a-5p Fw	GGCGGTGGCAGTGTCTTAGC	65
RT-mmu-miR-155-5p-STEM	GTCTCCTCTGGTGCAGGGTCCGAGGTATTCGCACCAG AGGAGACACCCCT	--
RT-mmu-miR-155-5p Fw	GCGGGCGGTTAATGCTAATTGTG	67
RT-mmu-miR-221-3p-STEM	GTCTCCTCTGGTGCAGGGTCCGAGGTATTCGCACCAG AGGAGACGAAACC	--
RT-mmu-miR-221-3p Fw	GGCGGAGCTACATTGTCTGCTG	67
RT-mmu-miR-138-5p-STEM	GTCTCCTCTGGTGCAGGGTCCGAGGTATTCGCACCAG AGGAGACCGGCCT	--
RT-mmu-miR-138-5p Fw	GGCGGAGCTGGTGTTGTGAATC	67
RT-mmu-miR-143-3p-STEM	GTCTCCTCTGGTGCAGGGTCCGAGGTATTCGCACCAG AGGAGACGAGCTA	--
RT-mmu-miR-143-3p Fw	GGGCGGTGAGATGAAGCACTG	65
RT-mmu-miR-27a-3p-STEM	GTCTCCTCTGGTGCAGGGTCCGAGGTATTCGCACCAG AGGAGACGCGGAA	--
RT-mmu-miR-27a-3p Fw	GGGCGGTTCACAGTGGCTAAG	70
RT-mmu-miR-125b-5p-STEM	GTCTCCTCTGGTGCAGGGTCCGAGGTATTCGCACCAG AGGAGACTCACAA	--
RT-mmu-miR-125b-5p Fw	CCGCCTCCCTGAGACCCTAAC	65
RT-mmu-miR-140-5p-STEM	GTCTCCTCTGGTGCAGGGTCCGAGGTATTCGCACCAG AGGAGACCTACCA	--
RT-mmu-miR-140-5p Fw	CGGCGGCAGTGGTTTTACCC	65
RT-mmu-miR-103a-3p-STEM	GTCTCCTCTGGTGCAGGGTCCGAGGTATTCGCACCAG AGGAGACTCATAG	--
RT-mmu-miR-103a-3p-Fw:	GGGGAGCAGCATTGTACAGGG	67
RT-mmu-miR-191-5p-STEM	GTCTCCTCTGGTGCAGGGTCCGAGGTATTCGCACCAG AGGAGACCAGCTG	--
RT-mmu-miR-191-5p Fw	GCGGCAACGGAATCCCAAAAG	70
RT- mmu-miR-19b-3p STEM	GTCTCCTCTGGTGCAGGGTCCGAGGTATTCGCACCAG AGGAGACTCAGTT	--
RT- mmu-miR-19b-3p Fw L	GGCGGTGTGCAAATCCATGC	65
RT-miR-Cel39-STEM	GTCTCCTCTGGTGCAGGGTCCGAGGTATTCGCACCAG AGGAGACCAAGCT	--
RT-miR-Cel39 Fw	CGGGGTCACCGGGTGTAAATC	65
