# Supplementary material for: Adipose tissue from metabolic syndrome mice induces an aberrant miRNA signature highly relevant in prostate cancer development
Source: Mol Oncol. 2020 Sep 25;14(11):2868–83. doi: 10.1002/1878-0261.12788 (PMC7607170; doi:10.1002/1878-0261.12788)
Supplement: Supplementary file 2 — Table S2. Clinical‐pathological data of patients. [file MOL2-14-2868-s002.pdf]

Table S2. Clinical-pathological data of patients

bcr_patient_bar code	Form completiondate	Histologic diagnosis	Gleason pattern primary	Gleason pattern secondary	Gleason score
TCGA-CH-5761	16/3/2012	Prostate Adenocarcinoma Acinar Type	5	4	9
TCGA-CH-5767	16/3/2012	Prostate Adenocarcinoma Acinar Type	4	3	7
TCGA-CH-5768	16/3/2012	Prostate Adenocarcinoma Acinar Type	2	4	6
TCGA-CH-5769	16/3/2012	Prostate Adenocarcinoma Acinar Type	5	4	9
TCGA-EJ-7115	5/4/2012	Prostate Adenocarcinoma Acinar Type	4	3	7
TCGA-EJ-7123	5/4/2012	Prostate Adenocarcinoma Acinar Type	3	4	7
TCGA-EJ-7125	5/4/2012	Prostate Adenocarcinoma Acinar Type	3	4	7
TCGA-EJ-7314	5/4/2012	Prostate Adenocarcinoma Acinar Type	4	3	7
TCGA-EJ-7315	5/4/2012	Prostate Adenocarcinoma Acinar Type	4	3	7
TCGA-EJ-7317	5/4/2012	Prostate Adenocarcinoma Acinar Type	3	4	7
TCGA-EJ-7321	5/4/2012	Prostate Adenocarcinoma Acinar Type	3	3	6
TCGA-EJ-7327	5/4/2012	Prostate Adenocarcinoma Acinar Type	3	4	7
TCGA-EJ-7328	5/4/2012	Prostate Adenocarcinoma Acinar Type	4	3	7
TCGA-EJ-7330	5/4/2012	Prostate Adenocarcinoma Acinar Type	4	3	7
TCGA-EJ-7331	5/4/2012	Prostate Adenocarcinoma Acinar Type	3	4	7
TCGA-EJ-7781	6/4/2012	Prostate Adenocarcinoma Acinar Type	3	4	7
TCGA-EJ-7782	6/4/2012	Prostate Adenocarcinoma Acinar Type	4	4	8
TCGA-EJ-7783	6/4/2012	Prostate Adenocarcinoma Acinar Type	4	3	7
TCGA-EJ-7784	6/4/2012	Prostate Adenocarcinoma Acinar Type	4	3	7
TCGA-EJ-7785	6/4/2012	Prostate Adenocarcinoma Acinar Type	3	4	7
TCGA-EJ-7786	6/4/2012	Prostate Adenocarcinoma Acinar Type	3	4	7
TCGA-EJ-7789	6/4/2012	Prostate Adenocarcinoma Acinar Type	4	3	7
TCGA-EJ-7792	9/4/2012	Prostate Adenocarcinoma Acinar Type	3	4	7
TCGA-EJ-7793	9/4/2012	Prostate Adenocarcinoma Acinar Type	3	4	7
TCGA-EJ-7794	9/4/2012	Prostate Adenocarcinoma Acinar Type	3	4	7
TCGA-EJ-7797	9/4/2012	Prostate Adenocarcinoma Acinar Type	3	4	7
TCGA-EJ-A8FO	24/3/2014	Prostate Adenocarcinoma, Other Subtype	4	3	7
TCGA-G9-6333	19/3/2012	Prostate Adenocarcinoma Acinar Type	4	3	7
TCGA-G9-6342	16/3/2012	Prostate Adenocarcinoma Acinar Type	3	3	6
TCGA-G9-6348	15/3/2012	Prostate Adenocarcinoma Acinar Type	3	4	7
TCGA-G9-6351	14/3/2012	Prostate Adenocarcinoma Acinar Type	3	4	7
TCGA-G9-6356	14/3/2012	Prostate Adenocarcinoma Acinar Type	4	5	9
TCGA-G9-6362	13/3/2012	Prostate Adenocarcinoma Acinar Type	4	3	7
TCGA-G9-6363	9/3/2012	Prostate Adenocarcinoma Acinar Type	4	3	7
TCGA-G9-6365	9/3/2012	Prostate Adenocarcinoma Acinar Type	3	4	7
TCGA-G9-6384	2/3/2012	Prostate Adenocarcinoma Acinar Type	3	4	7
TCGA-G9-6496	21/3/2012	Prostate Adenocarcinoma Acinar Type	4	3	7
TCGA-G9-6499	20/3/2012	Prostate Adenocarcinoma Acinar Type	4	5	9
TCGA-HC-7211	11/4/2012	Prostate Adenocarcinoma Acinar Type	3	4	7
TCGA-HC-7737	11/4/2012	Prostate Adenocarcinoma Acinar Type	3	4	7
TCGA-HC-7738	29/3/2012	Prostate Adenocarcinoma Acinar Type	3	4	7
TCGA-HC-7740	11/4/2012	Prostate Adenocarcinoma Acinar Type	3	4	7
TCGA-HC-7742	11/4/2012	Prostate Adenocarcinoma Acinar Type	3	4	7
TCGA-HC-7745	11/4/2012	Prostate Adenocarcinoma Acinar Type	4	3	7
TCGA-HC-7747	12/4/2012	Prostate Adenocarcinoma Acinar Type	3	4	7
TCGA-HC-7752	20/4/2012	Prostate Adenocarcinoma Acinar Type	3	4	7
TCGA-HC-7819	20/4/2012	Prostate Adenocarcinoma Acinar Type	4	4	8
TCGA-HC-8258	20/6/2012	Prostate Adenocarcinoma Acinar Type	3	3	6
TCGA-HC-8259	20/6/2012	Prostate Adenocarcinoma Acinar Type	3	3	6
TCGA-HC-8260	20/6/2012	Prostate Adenocarcinoma Acinar Type	3	4	7
TCGA-HC-8262	20/6/2012	Prostate Adenocarcinoma Acinar Type	4	4	8
TCGA-J4-A83J	20/1/2014	Prostate Adenocarcinoma Acinar Type	3	4	7

Laterality	Tumor level	Gender	Prospective collection	Retrospective collection	Birth days to	History other malignancy	History neoadjuvant treatment
Left	[Not Available]	MALE	YES	NO	-22433	No	No
Bilateral	[Not Available]	MALE	YES	NO	-24287	No	No
Bilateral	[Not Available]	MALE	YES	NO	-26480	No	No
Bilateral	[Not Available]	MALE	YES	NO	-17807	No	No
Bilateral	[Not Available]	MALE	YES	NO	-23986	No	No
Bilateral	Apex|Middle|Base	MALE	YES	NO	-21741	Yes	No
Bilateral	Apex|Middle	MALE	YES	NO	-16425	No	No
Bilateral	Apex|Middle|Base	MALE	YES	NO	-22766	No	No
Bilateral	Apex|Middle|Base	MALE	YES	NO	-25154	No	No
Bilateral	Apex|Middle	MALE	YES	NO	-26064	No	No
Bilateral	[Not Available]	MALE	YES	NO	-21108	No	No
Bilateral	Apex|Middle|Base	MALE	YES	NO	-22475	No	No
Bilateral	Middle|Base	MALE	YES	NO	-25756	No	No
Bilateral	Apex|Middle|Base	MALE	YES	NO	-25050	No	No
Bilateral	Apex|Middle	MALE	YES	NO	-23458	No	No
Bilateral	[Not Available]	MALE	YES	NO	-23937	No	No
Bilateral	[Not Available]	MALE	YES	NO	-25965	No	No
Bilateral	[Not Available]	MALE	YES	NO	-25600	No	No
Bilateral	[Not Available]	MALE	YES	NO	-23183	No	No
Bilateral	Middle|Base	MALE	YES	NO	-19939	No	No
Bilateral	[Not Available]	MALE	YES	NO	-22793	No	No
Bilateral	Base	MALE	YES	NO	-24107	No	No
Bilateral	Apex|Middle	MALE	YES	NO	-19454	No	No
Bilateral	Apex|Middle	MALE	YES	NO	-17982	No	No
Bilateral	[Not Available]	MALE	YES	NO	-24478	No	No
Bilateral	[Not Available]	MALE	YES	NO	-19616	No	No
Bilateral	[Not Available]	MALE	YES	NO	-18875	No	No
Bilateral	Apex|Base	MALE	NO	YES	-24249	No	No
Bilateral	Apex|Base	MALE	NO	YES	-22472	No	No
Bilateral	Apex|Base	MALE	NO	YES	-24936	No	No
Bilateral	Apex	MALE	NO	YES	-18723	No	No
Bilateral	Apex|Base	MALE	NO	YES	-22010	No	No
Bilateral	Apex|Base	MALE	NO	YES	-21149	No	No
Bilateral	Base	MALE	NO	YES	-23480	No	No
Bilateral	Apex|Base	MALE	NO	YES	-26207	No	No
Bilateral	Apex|Base	MALE	NO	YES	-19620	No	No
Bilateral	Apex|Base	MALE	NO	YES	-22602	No	No
Bilateral	Apex|Base	MALE	NO	YES	-24388	No	No
Bilateral	Apex|Middle|Base	MALE	NO	YES	-22768	No	No
Bilateral	[Not Available]	MALE	NO	YES	-20122	No	No
Bilateral	Apex	MALE	YES	NO	-21195	No	No
Bilateral	Apex|Middle|Base	MALE	YES	NO	-21883	No	No
[Not Available]	[Not Available]	MALE	YES	NO	-21323	No	No
Bilateral	Middle	MALE	YES	NO	-24078	No	No
Bilateral	Apex|Middle|Base	MALE	YES	NO	-19188	No	No
Bilateral	Base	MALE	YES	NO	-22366	No	No
Bilateral	[Not Available]	MALE	YES	NO	-19663	No	No
Bilateral	[Not Available]	MALE	YES	NO	-20703	No	No
Left	[Not Available]	MALE	YES	NO	-18309	No	No
Bilateral	Apex	MALE	YES	NO	-15884	No	No
Bilateral	[Not Available]	MALE	YES	NO	-20946	No	No
Bilateral	Apex|Middle|Base	MALE	YES	NO	-24960	No	No

Initial pathologic dx year	Days to bone scan	Bone scan results	Ct scan ab pelvis indicator	Days to ct scan ab pelvis
2007	28	Normal (no evidence of prostate cancer) [cM0]	YES	28
2007	[Not Available]	[Not Available]	NO	[Not Available]
2007	30	Normal (no evidence of prostate cancer) [cM0]	YES	30
2007	62	Normal (no evidence of prostate cancer) [cM0]	YES	62
2006	10	Normal (no evidence of prostate cancer) [cM0]	YES	10
2007	[Not Available]	[Not Available]	NO	[Not Available]
2005	12	Normal (no evidence of prostate cancer) [cM0]	NO	[Not Available]
2011	[Not Available]	[Not Available]	YES	14
2011	[Not Available]	[Not Available]	NO	[Not Available]
2011	[Not Available]	[Not Available]	[Not Available]	[Not Available]
2011	10	Normal (no evidence of prostate cancer) [cM0]	NO	[Not Available]
2011	[Not Available]	[Not Available]	NO	[Not Available]
2011	[Not Available]	[Not Available]	NO	[Not Available]
2011	[Not Available]	[Not Available]	NO	[Not Available]
2011	[Not Available]	[Not Available]	NO	[Not Available]
2011	[Not Available]	[Not Available]	NO	[Not Available]
2010	49	Normal (no evidence of prostate cancer) [cM0]	NO	[Not Available]
2011	[Not Available]	[Not Available]	NO	[Not Available]
2011	0	Normal (no evidence of prostate cancer) [cM0]	YES	0
2011	[Not Available]	[Not Available]	NO	[Not Available]
2011	[Not Available]	[Not Available]	NO	[Not Available]
2011	[Not Available]	[Not Available]	NO	[Not Available]
2010	[Not Available]	[Not Available]	NO	[Not Available]
2011	[Not Available]	[Not Available]	NO	[Not Available]
2010	35	Normal (no evidence of prostate cancer) [cM0]	YES	35
2011	[Not Available]	[Not Available]	NO	[Not Available]
2013	[Not Available]	[Not Available]	[Not Available]	[Not Available]
2006	1102	Normal (no evidence of prostate cancer) [cM0]	YES	1102
2008	32	Normal (no evidence of prostate cancer) [cM0]	YES	36
2008	31	Abnormal (not related to prostate cancer)	YES	31
2008	8	Normal (no evidence of prostate cancer) [cM0]	YES	11
2009	24	Normal (no evidence of prostate cancer) [cM0]	YES	21
2009	8	Normal (no evidence of prostate cancer) [cM0]	NO	[Not Available]
2009	58	Normal (no evidence of prostate cancer) [cM0]	YES	23
2009	[Not Available]	[Not Available]	NO	[Not Available]
2010	[Not Available]	[Not Available]	NO	[Not Available]
2008	19	Normal (no evidence of prostate cancer) [cM0]	YES	20
2008	56	Equivocal	YES	421
2010	14	Normal (no evidence of prostate cancer) [cM0]	YES	14
2010	9	Normal (no evidence of prostate cancer) [cM0]	YES	9
2011	[Not Available]	[Not Available]	[Not Available]	[Not Available]
2011	[Not Available]	[Not Available]	[Not Available]	[Not Available]
2011	[Not Available]	[Not Available]	[Not Available]	[Not Available]
2011	[Not Available]	[Not Available]	NO	[Not Available]
2011	25	Normal (no evidence of prostate cancer) [cM0]	YES	25
2011	[Not Available]	[Not Available]	[Not Available]	[Not Available]
2011	[Not Available]	[Not Available]	NO	[Not Available]
2011	[Not Available]	[Not Available]	[Not Available]	[Not Available]
2011	[Not Available]	[Not Available]	[Not Available]	[Not Available]
2011	[Not Available]	[Not Available]	[Not Available]	[Not Available]
2011	[Not Available]	[Not Available]	[Not Available]	[Not Available]
2013	[Not Available]	[Not Available]	[Not Available]	[Not Available]

Ct scan ab pelvis results	Mri at diagnosis	Lymph nodes examined	Lymph nodes examined count	Lymph nodes examined he count	Residual tumor
Equivocal	YES	YES	5	0	R0
[Not Available]	NO	YES	19	0	R0
No Evidence of Extraprostatic Extension	NO	YES	16	0	R0
No Evidence of Extraprostatic Extension	NO	YES	8	0	R1
No Evidence of Extraprostatic Extension	NO	YES	21	0	R0
[Not Available]	NO	YES	22	0	R0
[Not Available]	NO	YES	13	0	R0
No Evidence of Extraprostatic Extension	NO	YES	14	0	R0
[Not Available]	NO	YES	7	0	R0
[Not Available]	[Not Available]	YES	40	0	R0
[Not Available]	NO	YES	25	0	R0
[Not Available]	NO	YES	17	0	R0
[Not Available]	NO	YES	9	0	R0
[Not Available]	NO	YES	18	0	R0
[Not Available]	NO	YES	7	0	R0
[Not Available]	NO	YES	17	0	R0
[Not Available]	NO	YES	17	0	R0
[Not Available]	NO	YES	17	0	R0
No Evidence of Extraprostatic Extension	NO	YES	13	0	R0
[Not Available]	NO	YES	7	0	R0
[Not Available]	NO	YES	5	0	R0
[Not Available]	NO	YES	14	0	R0
[Not Available]	NO	YES	35	0	R0
[Not Available]	NO	NO	[Not Available]	[Not Available]	R0
No Evidence of Extraprostatic Extension	NO	YES	11	0	R0
[Not Available]	NO	YES	14	0	R0
[Not Available]	[Not Available]	YES	37	0	R0
No Evidence of Extraprostatic Extension	NO	YES	13	0	R0
No Evidence of Extraprostatic Extension	NO	NO	[Not Available]	[Not Available]	R0
No Evidence of Extraprostatic Extension	NO	[Discrepanc	15	0	R0
No Evidence of Extraprostatic Extension	NO	NO	[Not Available]	[Not Available]	R1
No Evidence of Extraprostatic Extension	NO	YES	5	0	R1
[Not Available]	NO	YES	2	0	R1
No Evidence of Extraprostatic Extension	NO	YES	6	0	R0
[Not Available]	NO	YES	13	0	R1
[Not Available]	NO	YES	14	0	R0
No Evidence of Extraprostatic Extension	NO	YES	15	0	R0
No Evidence of Extraprostatic Extension	NO	YES	13	0	R0
No Evidence of Extraprostatic Extension	NO	YES	10	0	R0
No Evidence of Extraprostatic Extension	NO	YES	8	1	R0
[Not Available]	[Not Available]	NO	[Not Available]	[Not Available]	R0
[Not Available]	[Not Available]	YES	4	0	R0
[Not Available]	[Not Available]	YES	2	0	R0
[Not Available]	NO	YES	3	0	R0
No Evidence of Extraprostatic Extension	NO	YES	4	0	[Not Available]
[Not Available]	[Not Available]	YES	6	0	R0
[Not Available]	YES	YES	3	0	R2
[Not Available]	[Not Available]	YES	2	0	[Not Available]
[Not Available]	[Not Available]	NO	[Not Available]	[Not Available]	R0
[Not Available]	[Not Available]	YES	9	0	R1
[Not Available]	[Not Available]	YES	8	0	R0
[Not Available]	[Not Available]	YES	1	0	R1

Vital status	Last contact days to	Tumor status	Days to psa most recent	Psa most recent results	Biochemical recurrence indicator	Age at initial pathologic diagnosis	Clinical M
Alive	28	TUMOR FREE	39	8.49	NO	61	M0
Alive	458	TUMOR FREE	377	0.04	NO	66	M0
Alive	731	TUMOR FREE	634	0.1	NO	72	M0
Alive	62	WITH TUMOR	60	11.16	NO	48	M0
Alive	822	TUMOR FREE	204	0.1	NO	65	M0
Alive	1491	TUMOR FREE	1491	0.1	NO	59	M0
Alive	1860	TUMOR FREE	1860	0.1	NO	44	M0
Alive	295	TUMOR FREE	295	0.1	NO	62	M0
Alive	289	TUMOR FREE	289	0.1	NO	68	M0
Alive	285	TUMOR FREE	285	0.1	NO	71	M0
Alive	233	TUMOR FREE	128	0.1	NO	57	M0
Alive	175	TUMOR FREE	175	0.1	NO	61	M0
Alive	170	TUMOR FREE	[Not Available]	[Not Available]	[Not Available]	70	M0
Alive	191	TUMOR FREE	191	0.1	NO	68	M0
Alive	9	TUMOR FREE	189	0.8	YES	64	M0
Alive	169	TUMOR FREE	169	0.1	NO	65	M0
Alive	350	TUMOR FREE	350	0.1	NO	71	M0
Alive	172	TUMOR FREE	172	0.7	YES	70	M0
Alive	167	TUMOR FREE	167	0.1	NO	63	M0
Alive	259	TUMOR FREE	259	0.1	NO	54	M0
Alive	79	TUMOR FREE	79	0.1	NO	62	M0
Alive	70	TUMOR FREE	[Not Available]	[Not Available]	[Not Available]	66	M0
Alive	526	TUMOR FREE	526	0.13	NO	53	M0
Alive	114	TUMOR FREE	114	0.1	NO	49	M0
Alive	285	TUMOR FREE	285	0.1	NO	67	M0
Alive	187	TUMOR FREE	187	0.1	NO	53	M0
Alive	156	TUMOR FREE	156	0.1	NO	51	M0
Alive	1925	TUMOR FREE	1920	0.03	NO	66	M0
Alive	1338	TUMOR FREE	1338	0.05	NO	61	M0
Alive	1144	TUMOR FREE	1136	0.02	NO	68	M0
Alive	1215	TUMOR FREE	1215	0.03	NO	51	M0
Alive	1000	TUMOR FREE	986	0.13	NO	60	M0
Alive	822	TUMOR FREE	815	0.07	NO	57	M0
Alive	993	TUMOR FREE	993	0.03	NO	64	M0
Alive	804	TUMOR FREE	804	0.01	NO	71	[Not Available]
Alive	390	TUMOR FREE	390	0.03	NO	53	M0
Alive	1014	TUMOR FREE	703	0.03	NO	61	M0
Alive	1109	TUMOR FREE	1109	0.11	NO	66	M0
Alive	24	TUMOR FREE	-17	10.9	[Not Available]	62	M0
Alive	64	TUMOR FREE	147	0.1	[Not Available]	55	M0
Alive	81	TUMOR FREE	[Not Available]	[Not Available]	[Not Available]	58	M0
Alive	53	TUMOR FREE	154	0	[Not Available]	59	M0
Alive	113	WITH TUMOR	113	0.1	NO	58	M0
Alive	63	TUMOR FREE	[Not Available]	[Not Available]	[Not Available]	65	[Not Available]
Alive	57	TUMOR FREE	392	0.19	NO	52	M0
Alive	98	TUMOR FREE	138	0.1	[Not Available]	61	M0
Alive	1	WITH TUMOR	172	0.01	[Not Available]	53	M0
Alive	66	TUMOR FREE	162	0.01	NO	56	[Not Available]
Alive	123	TUMOR FREE	123	0.01	NO	50	M0
Alive	106	WITH TUMOR	106	0.01	NO	43	M0
Alive	76	TUMOR FREE	112	0.1	NO	57	[Not Available]
Alive	310	[Unknown]	235	0.04	NO	68	M0

Clinical T	Ethnicity	Icd 10	Icd o 3 histology	Icd o 3 site	Informed consent verified	Initial pathologic diagnosis method
[Unknown]	NOT HISPANIC OR LATINO	C61	8550/3	C61.9	YES	Core needle biopsy
[Unknown]	NOT HISPANIC OR LATINO	C61	8550/3	C61.9	YES	Core needle biopsy
[Unknown]	NOT HISPANIC OR LATINO	C61	8550/3	C61.9	YES	Core needle biopsy
[Unknown]	NOT HISPANIC OR LATINO	C61	8550/3	C61.9	YES	Core needle biopsy
T1c	NOT HISPANIC OR LATINO	C61	8550/3	C61.9	YES	Core needle biopsy
T1c	NOT HISPANIC OR LATINO	C61	8550/3	C61.9	YES	Core needle biopsy
T1c	NOT HISPANIC OR LATINO	C61	8550/3	C61.9	YES	Core needle biopsy
T2c	NOT HISPANIC OR LATINO	C61	8550/3	C61.9	YES	Core needle biopsy
T2a	NOT HISPANIC OR LATINO	C61	8550/3	C61.9	YES	Core needle biopsy
T1c	NOT HISPANIC OR LATINO	C61	8550/3	C61.9	YES	Core needle biopsy
T1c	NOT HISPANIC OR LATINO	C61	8550/3	C61.9	YES	Core needle biopsy
T2a	NOT HISPANIC OR LATINO	C61	8550/3	C61.9	YES	Core needle biopsy
T1c	NOT HISPANIC OR LATINO	C61	8550/3	C61.9	YES	Core needle biopsy
T1c	NOT HISPANIC OR LATINO	C61	8550/3	C61.9	YES	Core needle biopsy
T1c	NOT HISPANIC OR LATINO	C61	8550/3	C61.9	YES	Core needle biopsy
T1c	NOT HISPANIC OR LATINO	C61	8550/3	C61.9	YES	Core needle biopsy
T2c	NOT HISPANIC OR LATINO	C61	8550/3	C61.9	YES	Core needle biopsy
T2c	NOT HISPANIC OR LATINO	C61	8550/3	C61.9	YES	Core needle biopsy
T2c	NOT HISPANIC OR LATINO	C61	8550/3	C61.9	YES	Core needle biopsy
T2b	NOT HISPANIC OR LATINO	C61	8550/3	C61.9	YES	Core needle biopsy
T1c	NOT HISPANIC OR LATINO	C61	8550/3	C61.9	YES	Core needle biopsy
T1c	NOT HISPANIC OR LATINO	C61	8550/3	C61.9	YES	Core needle biopsy
T1c	NOT HISPANIC OR LATINO	C61	8550/3	C61.9	YES	Core needle biopsy
T2c	NOT HISPANIC OR LATINO	C61	8550/3	C61.9	YES	Core needle biopsy
T2c	NOT HISPANIC OR LATINO	C61	8550/3	C61.9	YES	Core needle biopsy
T1c	NOT HISPANIC OR LATINO	C61	8550/3	C61.9	YES	Core needle biopsy
T3a	[Not Available]	C61	8500/3	C61.9	YES	Core needle biopsy
T1c	NOT HISPANIC OR LATINO	C61	8550/3	C61.9	YES	Core needle biopsy
T2c	NOT HISPANIC OR LATINO	C61	8550/3	C61.9	YES	Core needle biopsy
T3a	NOT HISPANIC OR LATINO	C61	8550/3	C61.9	YES	Core needle biopsy
T1c	NOT HISPANIC OR LATINO	C61	8550/3	C61.9	YES	Core needle biopsy
T1c	NOT HISPANIC OR LATINO	C61	8550/3	C61.9	YES	Core needle biopsy
T1c	NOT HISPANIC OR LATINO	C61	8550/3	C61.9	YES	Core needle biopsy
T2b	NOT HISPANIC OR LATINO	C61	8550/3	C61.9	YES	Core needle biopsy
T2a	NOT HISPANIC OR LATINO	C61	8550/3	C61.9	YES	Core needle biopsy
T1c	NOT HISPANIC OR LATINO	C61	8550/3	C61.9	YES	Core needle biopsy
T1c	NOT HISPANIC OR LATINO	C61	8550/3	C61.9	YES	Core needle biopsy
T3a	NOT HISPANIC OR LATINO	C61	8550/3	C61.9	YES	Core needle biopsy
T2a	NOT HISPANIC OR LATINO	C61	8550/3	C61.9	YES	Core needle biopsy
T1c	[Not Available]	C61	8550/3	C61.9	YES	Core needle biopsy
[Not Available]	NOT HISPANIC OR LATINO	C61	8550/3	C61.9	YES	Core needle biopsy
T2a	NOT HISPANIC OR LATINO	C61	8550/3	C61.9	YES	Core needle biopsy
[Unknown]	NOT HISPANIC OR LATINO	C61	8140/3	C61.9	YES	Core needle biopsy
[Unknown]	NOT HISPANIC OR LATINO	C61	8550/3	C61.9	YES	Core needle biopsy
T2b	NOT HISPANIC OR LATINO	C61	8550/3	C61.9	YES	Core needle biopsy
[Unknown]	NOT HISPANIC OR LATINO	C61	8550/3	C61.9	YES	Core needle biopsy
T2c	NOT HISPANIC OR LATINO	C61	8550/3	C61.9	YES	Core needle biopsy
[Unknown]	[Not Available]	C61	8550/3	C61.9	YES	Core needle biopsy
T1c	[Not Available]	C61	8550/3	C61.9	YES	Core needle biopsy
[Unknown]	[Not Available]	C61	8550/3	C61.9	YES	Core needle biopsy
[Unknown]	[Not Available]	C61	8550/3	C61.9	YES	Core needle biopsy
T1c	[Not Available]	C61	8140/3	C61.9	YES	Core needle biopsy

Pathologic N	Pathologic T	Patient id	Race	Tissue source site	Tumor tissue site
N0	T3b	5761	WHITE	CH	Prostate
N0	T2c	5767	WHITE	CH	Prostate
N0	T3a	5768	WHITE	CH	Prostate
N0	T3b	5769	WHITE	CH	Prostate
N0	T3a	7115	WHITE	EJ	Prostate
N0	T2c	7123	WHITE	EJ	Prostate
N0	T2c	7125	WHITE	EJ	Prostate
N0	T3b	7314	WHITE	EJ	Prostate
N0	T3a	7315	WHITE	EJ	Prostate
N0	T2c	7317	WHITE	EJ	Prostate
N0	T3a	7321	BLACK OR AFRICAN AMERICAN	EJ	Prostate
N0	T3b	7327	WHITE	EJ	Prostate
N0	T3a	7328	WHITE	EJ	Prostate
N0	T3a	7330	WHITE	EJ	Prostate
N0	T2c	7331	WHITE	EJ	Prostate
N0	T2c	7781	WHITE	EJ	Prostate
N0	T2c	7782	BLACK OR AFRICAN AMERICAN	EJ	Prostate
N0	T3a	7783	WHITE	EJ	Prostate
N0	T2c	7784	WHITE	EJ	Prostate
N0	T3a	7785	WHITE	EJ	Prostate
N0	T2c	7786	WHITE	EJ	Prostate
N0	T3a	7789	BLACK OR AFRICAN AMERICAN	EJ	Prostate
N0	T2c	7792	WHITE	EJ	Prostate
[Not Available]	T2c	7793	WHITE	EJ	Prostate
N0	T2c	7794	WHITE	EJ	Prostate
N0	T2c	7797	WHITE	EJ	Prostate
N0	T3a	A8FO	[Not Available]	EJ	Prostate
N0	T2c	6333	WHITE	G9	Prostate
[Not Available]	T3a	6342	WHITE	G9	Prostate
N0	T2c	6348	WHITE	G9	Prostate
[Not Available]	T2c	6351	WHITE	G9	Prostate
N0	T3b	6356	WHITE	G9	Prostate
N0	T3a	6362	WHITE	G9	Prostate
N0	T4	6363	WHITE	G9	Prostate
N0	T4	6365	WHITE	G9	Prostate
N0	T3a	6384	WHITE	G9	Prostate
N0	T2c	6496	WHITE	G9	Prostate
N0	T3a	6499	WHITE	G9	Prostate
N0	T2c	7211	WHITE	HC	Prostate
N1	T2c	7737	BLACK OR AFRICAN AMERICAN	HC	Prostate
[Not Available]	T2c	7738	WHITE	HC	Prostate
N0	T2c	7740	WHITE	HC	Prostate
N0	T3a	7742	WHITE	HC	Prostate
N0	T3b	7745	WHITE	HC	Prostate
N0	T2c	7747	WHITE	HC	Prostate
N0	T2c	7752	WHITE	HC	Prostate
N0	T2c	7819	WHITE	HC	Prostate
N0	T2c	8258	[Not Available]	HC	Prostate
[Not Available]	T2a	8259	[Not Available]	HC	Prostate
N0	T2c	8260	[Not Available]	HC	Prostate
N0	T2c	8262	[Not Available]	HC	Prostate
N0	T2c	A83J	[Not Available]	J4	Prostate
