# Supplementary material for: Adipose tissue from metabolic syndrome mice induces an aberrant miRNA signature highly relevant in prostate cancer development
Source: Mol Oncol. 2020 Sep 25;14(11):2868–83. doi: 10.1002/1878-0261.12788 (PMC7607170; doi:10.1002/1878-0261.12788)
Supplement: Supplementary file 3 — Table S3. Deregulated miRNAs. [file MOL2-14-2868-s003.pdf]

Table S3. All miRNAs with DE miRNAs highlighted in colors based on the relationship among FC and pvalue

Probe ID	Gene	log2FoldChange	pvalue	Probe ID	Gene	log2FoldChange	pvalue
MIMAT0003740	mmu-miR-674-5p	2.239353637	6.53E-06	MIMAT0009446	mmu-miR-1971	-0.761542297	0.009621534
MIMAT0027944	mmu-miR-7020-5p	1.594961419	0.00012398	MI0022814	mmu-mir-6966	-0.778432997	0.009579119
MIMAT0027876	mmu-miR-6987-5p	1.572350922	3.92E-05	MI0022777	mmu-mir-6930	-0.7906768	0.009344209
MIMAT0027860	mmu-miR-6979-5p	1.518168186	0.000185971	MIMAT0028007	mmu-miR-7051-3p	-0.828807927	0.009272431
MIMAT0000238	mmu-miR-205-5p	1.506190946	0.00078956	MIMAT0028439	mmu-miR-7235-3p	-0.780697118	0.009265905
MIMAT0003454	mmu-miR-423-3p	1.487865854	8.81E-05	MIMAT0004664	mmu-miR-214-5p	-0.954626039	0.009190865
MIMAT0027744	mmu-miR-6922-5p	1.379458255	0.00039478	MIMAT0017256	mmu-miR-700-5p	-0.940940842	0.008939641
MIMAT0000139	mmu-miR-127-3p	1.344865096	0.000711256	MIMAT0000225	mmu-miR-195a-5p	-0.91446158	0.008303426
MIMAT0027920	mmu-miR-7008-5p	1.301033524	0.000652529	MIMAT0020620	mmu-miR-5112	-0.90000442	0.00804894
MIMAT0000376	mmu-miR-298-5p	1.251862036	0.000858075	MIMAT0000537	mmu-miR-27a-3p	-0.900755618	0.008006525
MIMAT0028416	mmu-miR-7224-5p	1.241260029	0.001194127	MIMAT0027717	mmu-miR-6908-3p	-0.869044866	0.00771615
MIMAT0004871	mmu-miR-465b-5p	1.22968503	0.001145188	MIMAT0014863	mmu-miR-3077-3p	-0.841587061	0.007657422
MIMAT0000666	mmu-miR-320-3p	1.223942044	0.000861338	MIMAT0014929	mmu-miR-344g-5p	-0.899348589	0.007373573
MI0025015	mmu-mir-7674	1.189540838	0.001096248	MIMAT0000224	mmu-miR-194-5p	-0.833144149	0.007298532
MI0008327	mmu-miR-1893	1.185602947	0.001027733	MI0022827	mmu-mir-6979	-0.857963484	0.006649266
MIMAT0004528	mmu-miR-125a-3p	1.171284166	0.001070147	MIMAT0027705	mmu-miR-6902-3p	-0.886628259	0.006016314
MIMAT0000158	mmu-miR-146a-5p	1.160189848	0.002590539	MIMAT0029841	mmu-miR-7667-3p	-0.934850179	0.006003263
MIMAT0003189	mmu-miR-291b-5p	1.13969178	0.001230016	MIMAT0027746	mmu-miR-6923-5p	-0.923518281	0.005644372
MIMAT0000609	mmu-miR-351-5p	1.126147501	0.001810767	MIMAT0025091	mmu-miR-6348	-0.914907898	0.004933116
MIMAT0000661	mmu-miR-214-3p	1.125580281	0.001615008	MIMAT0003499	mmu-miR-709	-0.984916604	0.004822186
MIMAT0004526	mmu-miR-101a-5p	1.095148931	0.002792822	MIMAT0003494	mmu-miR-704	-0.989372145	0.004756933
MI0000704	mmu-mir-320	1.090064263	0.001784666	MIMAT0028419	mmu-miR-7225-3p	-0.966491304	0.004189233
MIMAT0014858	mmu-miR-3075-5p	1.055526224	0.001738989	MIMAT0027835	mmu-miR-6966-3p	-0.999007301	0.004120718
MIMAT0003725	mmu-miR-675-5p	1.046516613	0.002215334	MIMAT0028452	mmu-miR-7242-5p	-0.998846754	0.003389886
MIMAT0000650	mmu-miR-17-3p	1.03638768	0.007915172	MIMAT0027972	mmu-miR-7034-5p	-1.000153206	0.003301794
MI0026035	mmu-miR-8105	1.002217799	0.00245677	MIMAT0007872	mmu-miR-1906	-1.013175801	0.003389886
MIMAT0000367	mmu-miR-291a-5p	0.904615367	0.003324633	MIMAT0003735	mmu-miR-672-5p	-1.024413161	0.003598695
MIMAT0029868	mmu-miR-7677-5p	0.935963318	0.003389886	MIMAT0000157	mmu-miR-145a-5p	-1.02683879	0.00702447
MIMAT0027812	mmu-miR-6956-5p	0.913874427	0.00348124	MIMAT0029861	mmu-miR-7676-3p	-1.052128718	0.004362153
MIMAT0004535	mmu-miR-150-3p	0.927054284	0.003513866	MIMAT0017071	mmu-miR-7b-3p	-1.062819744	0.002623165
MIMAT0028008	mmu-miR-7052-5p	0.899369986	0.003879282	MIMAT0003711	mmu-miR-652-3p	-1.083602324	0.001921044
MIMAT0000214	mmu-miR-185-5p	0.876130953	0.004208809	MIMAT0004750	mmu-miR-425-5p	-1.099163891	0.001993148
MIMAT0027770	mmu-miR-6935-5p	0.933810212	0.004414355	MIMAT0028038	mmu-miR-7067-5p	-1.107943381	0.002698206
MIMAT0000670	mmu-miR-222-3p	0.764663103	0.004854812	MIMAT0027943	mmu-miR-7019-3p	-1.147711118	0.002101142
MI0021877	mmu-miR-6349	0.845130302	0.004965742	MIMAT0027970	mmu-miR-7033-5p	-1.155880675	0.002854812
MIMAT0003496	mmu-miR-706	0.809222986	0.005194127	MI0022837	mmu-mir-6989	-1.191505169	0.001712887
MI0016973	mmu-miR-378b	0.970701764	0.005324633	MIMAT0028064	mmu-miR-7079-5p	-1.226270034	0.00184013
MIMAT0027914	mmu-miR-7005-5p	0.807641939	0.005425775	MIMAT0000136	mmu-miR-125b-5p	-1.22774816	0.003601958
MIMAT0004825	mmu-miR-423-5p	0.826739346	0.005644372	MIMAT0000531	mmu-miR-22-3p	-1.233723233	0.00258075
MIMAT0000590	mmu-miR-342-3p	0.57609982	0.005973899	MIMAT0017340	mmu-miR-1930-3p	-1.239416312	0.000916803
MIMAT0014915	mmu-miR-3097-5p	0.841012614	0.005990212	MIMAT0024857	mmu-miR-6236	-1.287507106	0.008786297
MIMAT0000669	mmu-miR-221-3p	0.942415062	0.006042414	MIMAT0015645	mmu-miR-3473a	-1.321993188	0.000822186
MI0022872	mmu-mir-7023	0.882276237	0.006104404	MIMAT0004888	mmu-miR-493-3p	-1.328010986	0.001615008
MIMAT0027868	mmu-miR-6983-5p	0.938896825	0.006274062	MIMAT0004656	mmu-miR-345-3p	-1.39300206	0.000655791
MI0022932	mmu-mir-7082	0.803465848	0.006593801	MIMAT0007877	mmu-miR-1894-5p	-1.400301778	0.000492659
MIMAT0027794	mmu-miR-6947-5p	0.764336163	0.006792822	MIMAT0000535	mmu-miR-29a-3p	-1.414135533	0.001122349
MIMAT0025138	mmu-miR-378c	0.943963135	0.006890702	MIMAT0025080	mmu-miR-6337	-1.425639657	0.000584013
MIMAT0014908	mmu-miR-3093-3p	0.895907747	0.006900489	MIMAT0031396	mmu-miR-8095	-1.484043392	0.000613377
MIMAT0028076	mmu-miR-7085-5p	0.745266169	0.007096248	MIMAT0028003	mmu-miR-7049-3p	-1.485560682	0.000681892
MIMAT0031413	mmu-miR-8108	0.968855533	0.007461664	MIMAT0000538	mmu-miR-31-5p	-1.520925815	0.000512235
MIMAT0029815	mmu-miR-7654-3p	0.728021974	0.007833606	MIMAT0014834	mmu-miR-3064-5p	-1.585564075	0.000313214
MIMAT0027912	mmu-miR-7004-5p	0.772112198	0.008035889	MIMAT0027980	mmu-miR-7038-5p	-1.605126478	0.000185971
MI0026041	mmu-miR-8109	0.910610137	0.008133768	MIMAT0025123	mmu-let-7j	-1.605543403	0.000179445
MIMAT0003486	mmu-miR-491-5p	0.922504586	0.008522023	MIMAT0028050	mmu-miR-7072-5p	-1.650228963	0.00034584
MIMAT0003733	mmu-miR-665-3p	0.855275397	0.008570963	MIMAT0031390	mmu-miR-3473f	-1.65583663	0.000652529
MIMAT0004576	mmu-miR-296-3p	0.805285966	0.008903752	MIMAT0017050	mmu-miR-32-3p	-1.697389791	0.000492659
MIMAT0017002	mmu-miR-204-3p	0.999522662	0.009125612	MIMAT0004828	mmu-miR-708-5p	-1.854819684	6.85E-05
				MIMAT0000649	mmu-miR-17-5p	-1.900012267	0.004303426
				MIMAT0000647	mmu-miR-107-3p	-2.082639751	0.001621533
				MIMAT0000529	mmu-miR-20a-5p	-2.190298065	0.000358891
				MIMAT0000546	mmu-miR-103-3p	-2.233359834	0.001709625
				MIMAT0000533	mmu-miR-26a-5p	-2.2961449	8.81E-05
				MIMAT0000539	mmu-miR-92a-3p	-2.296568854	0.000502447
				MIMAT0000527	mmu-miR-16-5p	-2.530279164	0.001057096
