# Supplementary material for: Adipose tissue from metabolic syndrome mice induces an aberrant miRNA signature highly relevant in prostate cancer development
Source: Mol Oncol. 2020 Sep 25;14(11):2868–83. doi: 10.1002/1878-0261.12788 (PMC7607170; doi:10.1002/1878-0261.12788)
Supplement: Supplementary file 4 — Table S4. miRNAs with LOGFC>1, pvalue<0.05 and their targets. [file MOL2-14-2868-s004.pdf]

Table S4. Lists of DE miRNAs (LOGFC>1, pvalue<0.05) and their respective number of experimentally validated targets based on DIANA-TarBasev7

down modulated miRNA	# of validated targets
mmu-miR-22-3p	2150
mmu-miR-26a-5p	2092
mmu-miR-17-5p	2019
mmu-miR-16-5p	1968
mmu-miR-20a-5p	1778
mmu-miR-125b-5p	1624
mmu-miR-92a-3p	1373
mmu-miR-31-5p	1176
mmu-miR-708-5p	812
mmu-miR-29a-3p	547
mmu-miR-103-3p	332
mmu-miR-107-3p	311
mmu-miR-652-3p	68
mmu-miR-1894-5p	51
mmu-miR-493-3p	17
mmu-miR-345-3p	11
mmu-miR-672-5p	7
mmu-let-7j	0
mmu-miR-145a-5p	0
mmu-miR-1906	0
mmu-miR-1930-3p	0
mmu-miR-3064-5p	0
mmu-miR-32-3p	0
mmu-miR-3473a	0
mmu-miR-3473f	0
mmu-miR-6236	0
mmu-miR-6337	0
mmu-miR-6989-3p	0
mmu-miR-6989-5p	0
mmu-miR-7019-3p	0
mmu-miR-7033-5p	0
mmu-miR-7034-5p	0
mmu-miR-7038-5p	0
mmu-miR-7049-3p	0
mmu-miR-7067-5p	0
mmu-miR-7072-5p	0
mmu-miR-7079-5p	0
mmu-miR-7676-3p	0
mmu-miR-7b-3p	0
mmu-miR-8095	0

up modulated miRNA	#of validated targets
mmu-miR-125a-3p	967
mmu-miR-214-3p	479
mmu-miR-146a-5p	360
mmu-miR-320-3p	186
mmu-miR-320-3p	186
mmu-miR-101a-5p	103
mmu-miR-205-5p	64
mmu-miR-351-5p	27
mmu-miR-17-3p	18
mmu-miR-298-5p	15
mmu-miR-674-5p	14
mmu-miR-127-3p	4
mmu-miR-423-3p	4
mmu-miR-1893	0
mmu-miR-291b-5p	0
mmu-miR-3075-5p	0
mmu-miR-465b-5p	0
mmu-miR-675-5p	0
mmu-miR-6922-5p	0
mmu-miR-6979-5p	0
mmu-miR-6987-5p	0
mmu-miR-7008-5p	0
mmu-miR-7020-5p	0
mmu-miR-7224-5p	0
mmu-miR-7674-3p	0
mmu-miR-8105	0
