# Supplementary material for: Adipose tissue from metabolic syndrome mice induces an aberrant miRNA signature highly relevant in prostate cancer development
Source: Mol Oncol. 2020 Sep 25;14(11):2868–83. doi: 10.1002/1878-0261.12788 (PMC7607170; doi:10.1002/1878-0261.12788)
Supplement: Supplementary file 5 — Table S5. Functional enrichment of the down modulated miRNAs. [file MOL2-14-2868-s005.pdf]

Table S5. Functional enrichment of target genes for down modulated miRNAs. DIANA-miRPath v3 tool

#	KEGG pathway	p-value	#miRNAs	#genes	genes							
1	Fatty acid metabolism (mmu01212)	7.58E-11	12	**28**	Scd2	Scd1	Ppt1	Mcat	Hsd17b12	Hadha	Hadh	Fasn
	mmu-miR-16-5p			11	Fads2	Fads1	Elovl6	Elovl5	Elovl2	Ehhadh	Echs1	Cpt2
	mmu-miR-103-3p			6	Cpt1a	Acsl6	Acsl3	Acsl1	Acsbg1	Acox3	Acox1	Acat2
	mmu-miR-107-3p			6	Acat1	Acadsb	Acads	Acaa2				
	mmu-miR-20a-5p			7								
	mmu-miR-125b-5p			6								
	mmu-miR-31-5p			7								
	mmu-miR-26a-5p			7								
	mmu-miR-17-5p			10								
	mmu-miR-708-5p			6								
	mmu-miR-29a-3p			1								
	mmu-miR-92a-3p			1								
	mmu-miR-1894-5p			1								
2	Adherens junction (mmu04520)	1.47E-08	13	**48**	Wasl	Wasf2	Vcl	Tjp1	Tgfbr2	Tgfbr1	Tcf7l2	Tcf7l1
	mmu-miR-92a-3p			17	Tcf7	Ssx2ip	Src	Snai1	Smad4	Smad3	Smad2	Rac2
	mmu-miR-26a-5p			13	Rac1	Pvrl3	Pvrl2	Pvrl1	Ptprm	Ptprf	Ptprb	Ptpn1
	mmu-miR-20a-5p			19	Nlk	Mllt4	Met	Map3k7	Lmo7	Lef1	Iqgap1	Insr
	mmu-miR-17-5p			17	Igf1r	Fyn	Fgfr1	Fer	Erbb2	Ep300	Egfr	Ctnnd1
	mmu-miR-16-5p			14	Csnk2a1	Crebbp	Cdh1	Cdc42	Baiap2	Actn4	Actn2	Actb
	mmu-miR-31-5p			11								
	mmu-miR-708-5p			12								
	mmu-miR-103-3p			3								
	mmu-miR-107-3p			3								
	mmu-miR-125b-5p			18								
	mmu-miR-29a-3p			1								
	mmu-miR-652-3p			1								
	mmu-miR-672-5p			1								
3	Fatty acid degradation (mmu00071)	4.08E-08	11	**26**	Hadha	Hadh	Gcdh	Ehhadh	Echs1	Cyp4a14	Cyp4a12a	Cyp4a10
	mmu-miR-20a-5p			6	Cpt2	Cpt1a	Aldh7a1	Aldh3a2	Aldh2	Adh5	Adh4	Acsl6
	mmu-miR-17-5p			5	Acsl3	Acsl1	Acsbg1	Acox3	Acox1	Acat2	Acat1	Acadsb
	mmu-miR-31-5p			6	Acads	Acaa2						
	mmu-miR-92a-3p			2								
	mmu-miR-26a-5p			5								
	mmu-miR-125b-5p			3								
	mmu-miR-103-3p			6								
	mmu-miR-107-3p			6								
	mmu-miR-16-5p			6								
	mmu-miR-708-5p			4								
4	Proteoglycans in cancer (mmu05205)	2.21E-07	14	**105**	Wnt5a	Wnt2	Vtn	Vegfa	Vav2	Tlr4	Timp3	Tiam1
	mmu-miR-708-5p			12	Tgfb2	Tgfb1	Tfap4	Stat3	Src	Sos2	Sos1	Smad2
	mmu-miR-16-5p			38	Sdc4	Sdc2	Sdc1	Rras2	Rras	Rps6kb1	Rock2	Rock1
	mmu-miR-20a-5p			33	Rdx	Rac1	Ptpn11	Ptk2	Prkx	Prkcg	Prkcb	Prkca
	mmu-miR-31-5p			25	Prkacb	Prkaca	Ppp1r12c	Ppp1r12b	Ppp1r12a	Ppp1cb	Ppp1ca	Plce1
	mmu-miR-103-3p			6	Pik3r3	Pik3r2	Pik3r1	Pik3cd	Pik3cb	Pdpk1	Pak1	Nras
	mmu-miR-107-3p			6	Myc	Mtor	Mras	Met	Mdm2	Mapk14	Kras	Kdr
	mmu-miR-125b-5p			28	Itpr2	Itpr1	Itgb3	Itgb1	Itgav	Itga5	Iqgap1	Igf1r
	mmu-miR-92a-3p			29	Igf1	Hif1a	Hgf	Hbegf	Grb2	Gpc1	Gab1	Fzd7
	mmu-miR-26a-5p			37	Fzd5	Fzd1	Frs2	Fn1	Flnb	Fgfr1	Fasl	Ezr
	mmu-miR-29a-3p			16	Esr1	Erbb2	Elk1	Eif4b	Egfr	Ddx5	Ctsl	Col1a2
	mmu-miR-17-5p			37	Col1a1	Cdc42	Cd44	Ccnd1	Cblb	Casp3	Camk2g	Camk2d
	mmu-miR-652-3p			4	Camk2a	Braf	Arhgef12	Arhgef1	Araf	Ank3	Ank2	Akt1
	mmu-miR-493-3p			1	Actb							
	mmu-miR-22-3p			3								
5	N-Glycan biosynthesis (mmu00510)	1.34E-06	12	**26**	Stt3b	Stt3a	Rpn2	Rpn1	Mgat5	Mgat4b	Mgat3	Mgat2
	mmu-miR-26a-5p			10	Man2a2	Man2a1	Man1c1	Man1b1	Man1a2	Man1a	Ganab	Dpagt1
	mmu-miR-20a-5p			10	Dolk	B4galt2	B4galt1	Alg9	Alg6	Alg5	Alg2	Alg14
	mmu-miR-17-5p			9	Alg11	Alg10b						
	mmu-miR-125b-5p			7								
	mmu-miR-16-5p			11								
	mmu-miR-92a-3p			3								
	mmu-miR-29a-3p			1								
	mmu-miR-31-5p			4								
	mmu-miR-103-3p			1								
	mmu-miR-107-3p			1								
	mmu-miR-708-5p			5								
	mmu-miR-652-3p			1								
6	Protein processing in endoplasmic reticulum (mmu04141)	6.51E-06	12	**96**	Yod1	Xbp1	Wfs1	Vimp	Uggt2	Ubqln4	Ubqln2	Ubqln1
	mmu-miR-16-5p			36	Ube4b	Ube2j1	Ube2g2	Ube2g1	Ube2d3	Ube2d1	Txndc5	Tram1
	mmu-miR-708-5p			16	Svip	Stub1	Stt3b	Stt3a	Ssr4	Ssr3	Ssr1	Sil1
	mmu-miR-26a-5p			36	Sel1l	Sec63	Sec62	Sec61a1	Sec31a	Sec24d	Sec24c	Sec24b
	mmu-miR-20a-5p			26	Sec24a	Sec23b	Sec23a	Sar1b	Rrbp1	Rpn2	Rpn1	Rnf5
	mmu-miR-17-5p			25	Rad23b	Prkcsh	Preb	Plaa	Pdia6	Pdia4	Pdia3	Os9
	mmu-miR-29a-3p			7	Nploc4	Ngly1	Nfe2l2	Mbtps2	March6	Map3k5	Mapk9	Mapk8
	mmu-miR-125b-5p			29	Mapk10	Map2k7	Man1c1	Man1b1	Man1a2	Man1a	Lman2	Hspa5
	mmu-miR-652-3p			2	Hspa4l	Hsp90ab1	Hsp90aa1	Ganab	Erp29	Ero1lb	Ero1l	Ern1
	mmu-miR-103-3p			7	Eif2s1	Eif2ak4	Eif2ak3	Eif2ak2	Eif2ak1	Edem3	Edem1	Dnajc3
	mmu-miR-107-3p			6	Dnajc10	Dnajb12	Dnajb1	Dnaja2	Derl1	Cul1	Ckap4	Capn2
	mmu-miR-92a-3p			20	Canx	Calr	Bcl2	Bak1	Bag2	Atxn3	Atf6	Atf4
	mmu-miR-31-5p			16								
7	FoxO signaling pathway (mmu04068)	1.91E-05	12	**77**	Usp7	Tgfbr2	Tgfbr1	Tgfb3	Tgfb2	Tgfb1	Stk4	Stk11
	mmu-miR-20a-5p			32	Stat3	Sos2	Sos1	Sod2	Smad4	Smad3	Smad2	Skp2
	mmu-miR-17-5p			42	Sgk1	Setd7	S1pr4	S1pr1	Rbl2	Pten	Prmt1	Prkag2
	mmu-miR-31-5p			21	Prkab2	Prkaa2	Prkaa1	Plk3	Plk2	Pik3r3	Pik3r2	Pik3r1
	mmu-miR-92a-3p			32	Pik3cd	Pik3cb	Pdpk1	Pck1	Nras	Nlk	Mdm2	Mapk9
	mmu-miR-708-5p			11	Mapk8	Mapk14	Mapk10	Kras	Klf2	Irs1	Insr	Il7r
	mmu-miR-125b-5p			21	Il6	Igf1r	Igf1	Homer1	Grb2	Gabarapl1	G6pc	Foxo4
	mmu-miR-26a-5p			33	Fbxo32	Fbxo25	Fasl	Foxo1	Ep300	Egfr	Csnk1e	Crebbp
	mmu-miR-29a-3p			13	Chuk	Cdk2	Ccng2	Ccnd2	Ccnd1	Braf	Bcl6	Bcl2l11
	mmu-miR-16-5p			26	Atm	Atg12	Araf	Akt1	Agap2			
	mmu-miR-103-3p			3								
	mmu-miR-107-3p			3								
	mmu-miR-652-3p			1								
8	Lysine degradation (mmu00310)	3.08E-05	12	**30**	Whsc1l1	Whsc1	Suv420h1	Suv39h1	Setdb1	Setd8	Setd7	Setd2
	mmu-miR-103-3p			5	Setd1b	Setd1a	Plod2	Ogdh	Nsd1	Kmt2d	Kmt2c	Kmt2b
	mmu-miR-20a-5p			13	Hadha	Hadh	Gcdh	Ehmt1	Ehhadh	Echs1	Dot1l	Dlst
	mmu-miR-107-3p			5	Colgalt1	Aldh7a1	Aldh3a2	Aldh2	Acat2	Acat1		
	mmu-miR-17-5p			12								
	mmu-miR-16-5p			13								
	mmu-miR-26a-5p			11								
	mmu-miR-125b-5p			10								
	mmu-miR-31-5p			7								
	mmu-miR-29a-3p			4								
	mmu-miR-92a-3p			4								
	mmu-miR-708-5p			4								
	mmu-miR-493-3p			1								

9	Cell cycle (mmu04110)	3.91E-05	11	**67**	Zbtb17	Ywhah	Ywhag	Ywhae	Ywhab	Wee1	Tgfb3	Tgfb2
	mmu-miR-20a-5p			26	Tgfb1	Tfdp2	Tfdp1	Stag2	Stag1	Smc3	Smc1a	Smad4
	mmu-miR-17-5p			26	Smad3	Smad2	Skp2	Rbl2	Rbl1	Rb1	Rad21	Prkdc
	mmu-miR-16-5p			27	Orc4	Orc2	Myc	Mdm2	Mcm7	Mcm5	Mcm4	Mcm3
	mmu-miR-26a-5p			23	Hdac2	Gsk3b	Ep300	E2f5	E2f3	E2f2	E2f1	Cul1
	mmu-miR-31-5p			16	Crebbp	Chek2	Chek1	Cdk7	Cdk4	Cdk2	Cdc7	Cdc27
	mmu-miR-708-5p			7	Cdc25a	Cdc23	Cdc16	Cdc14b	Cdc14a	Ccne2	Ccne1	Ccnd1
	mmu-miR-29a-3p			13	Ccnd2	Ccna2	Bub3	Bub1	Atr	Atm	Anapc7	Anapc4
	mmu-miR-92a-3p			12	Anapc2	Anapc10	Anapc1					
	mmu-miR-125b-5p			9								
	mmu-miR-103-3p			2								
	mmu-miR-107-3p			2								
10	Hippo signaling pathway (mmu04390)	3.91E-05	13	**71**	Ywhah	Ywhag	Ywhae	Ywhab	Yap1	Wwtr1	Wnt5a	Wnt2
	mmu-miR-125b-5p			24	Trp53bp2	Tgfbr2	Tgfbr1	Tgfb3	Tgfb2	Tgfb1	Tead1	Tcf7l2
	mmu-miR-16-5p			28	Tcf7l1	Tcf7	Stk3	Smad7	Smad4	Smad3	Smad2	Smad1
	mmu-miR-26a-5p			21	Serpine1	Sav1	Prkcz	Prkci	Ppp2r2a	Ppp2r1b	Ppp2cb	Ppp2ca
	mmu-miR-20a-5p			26	Ppp1cb	Ppp1ca	Pard6a	Nf2	Myc	Mpp5	Mob1b	Mob1a
	mmu-miR-17-5p			37	Llgl1	Lef1	Lats2	Lats1	Itgb2	Id2	Id1	Gsk3b
	mmu-miR-92a-3p			15	Fzd7	Fzd5	Fzd1	Dvl3	Dvl1	Dlg4	Ctgf	Csnk1d
	mmu-miR-31-5p			15	Csnk1e	Crb2	Cdh1	Ccnd2	Ccnd1	Bmpr2	Bmpr1a	Bmp7
	mmu-miR-708-5p			6	Bmp4	Bbc3	Axin1	Apc	Amot	Ajuba	Actb	
	mmu-miR-103-3p			3								
	mmu-miR-107-3p			3								
	mmu-miR-493-3p			1								
	mmu-miR-29a-3p			6								
	mmu-miR-652-3p			1								
11	Fatty acid biosynthesis (mmu00061)	4.11E-05	6	**6**	Mcat	Fasn	Acsl6	Acsl3	Acsl1	Acsbg1		
	mmu-miR-17-5p			3								
	mmu-miR-16-5p			3								
	mmu-miR-103-3p			1								
	mmu-miR-107-3p			1								
	mmu-miR-20a-5p			1								
	mmu-miR-22-3p			1								
	mmu-miR-26a-5p			1								
12	Phosphatidylinositol signaling system (mmu04070)	4.70E-05	12	**49**	Synj2	Synj1	Pten	Prkcg	Prkcb	Prkca	Plce1	Plcd1
	mmu-miR-16-5p			15	Plcb1	Pip5k1c	Pip5k1a	Pip4k2c	Pip4k2b	Pip4k2a	Pikfyve	Pik3r3
	mmu-miR-20a-5p			13	Pik3r2	Pik3r1	Pik3cd	Pik3cb	Pik3c2b	Pik3c2a	Pi4kb	Pi4k2b
	mmu-miR-92a-3p			14	Pi4k2a	Mtm1	Itpr3	Itpr1	Itpkc	Itpkb	Itpk1	Ippk
	mmu-miR-26a-5p			15	Inpp5e	Inpp5d	Inpp5b	Inpp5a	Inpp4b	Inpp4a	Impad1	Impa2
	mmu-miR-17-5p			16	Dgkh	Dgke	Dgkd	Dgka	Cds2	Cdipt	Calm3	Calm2
	mmu-miR-31-5p			10	Calm1							
	mmu-miR-29a-3p			5								
	mmu-miR-125b-5p			16								
	mmu-miR-103-3p			3								
	mmu-miR-107-3p			3								
	mmu-miR-708-5p			5								
	mmu-miR-493-3p			1								
13	Central carbon metabolism in cancer (mmu05230)	0.000397114	11	**35**	Slc7a5	Slc2a1	Slc1a5	Sirt6	Sirt3	Ret	Pten	Pkm
	mmu-miR-16-5p			8	Pik3r3	Pik3r2	Pik3r1	Pik3cd	Pik3cb	Pgam1	Pfkp	Pdk1
	mmu-miR-31-5p			5	Pdhb	Pdha1	Pdgfrb	Nras	Myc	Mtor	Met	Ldha
	mmu-miR-29a-3p			5	Kras	Hk2	Hk1	Hif1a	Gls	Gck	Fgfr3	Fgfr1
	mmu-miR-92a-3p			9	Erbb2	Egfr	Akt1					
	mmu-miR-26a-5p			7								
	mmu-miR-17-5p			11								
	mmu-miR-125b-5p			10								
	mmu-miR-708-5p			4								
	mmu-miR-20a-5p			8								
	mmu-miR-103-3p			1								
	mmu-miR-107-3p			1								
14	Prostate cancer (mmu05215)	0.000505908	11	**51**	Tcf7l2	Tcf7l1	Tcf7	Sos2	Sos1	Rela	Rb1	Pten
	mmu-miR-31-5p			11	Pik3r3	Pik3r2	Pik3r1	Pik3cd	Pik3cb	Pdpk1	Pdgfrb	Pdgfb
	mmu-miR-26a-5p			25	Nras	Nfkbia	Nfkb1	Mtor	Mdm2	Lef1	Kras	Igf1r
	mmu-miR-20a-5p			21	Igf1	Hsp90ab1	Hsp90aa1	Gsk3b	Grb2	Foxo1	Fgfr1	Erbb2
	mmu-miR-17-5p			31	Ep300	Egfr	E2f3	E2f2	E2f1	Crebbp	Creb3l2	Creb1
	mmu-miR-708-5p			6	Chuk	Cdk2	Ccne2	Ccne1	Ccnd1	Braf	Bcl2	Atf4
	mmu-miR-29a-3p			10	Araf	Ar	Akt1					
	mmu-miR-125b-5p			13								
	mmu-miR-16-5p			17								
	mmu-miR-92a-3p			13								
	mmu-miR-103-3p			1								
	mmu-miR-22-3p			1								
	mmu-miR-1894-5p			1								
	mmu-miR-107-3p			1								
15	Valine, leucine and isoleucine degradation (mmu00280)	0.000856932	11	**28**	Pcca	Oxct1	Mut	Mcee	Hmgcs2	Hmgcs1	Hibadh	Hadha
	mmu-miR-92a-3p			3	Hadh	Gene Name	Ehhadh	Echs1	Dld	Dbt	Bckdha	Bcat1
	mmu-miR-26a-5p			6	Auh	Aox3	Aldh7a1	Aldh6a1	Aldh3a2	Aldh2	Acat2	Acat1
	mmu-miR-103-3p			4	Acadsb	Acads	Acaa2	Abat	Aacs			
	mmu-miR-107-3p			4								
	mmu-miR-31-5p			5								
	mmu-miR-16-5p			9								
	mmu-miR-708-5p			4								
	mmu-miR-17-5p			7								
	mmu-miR-20a-5p			6								
	mmu-miR-29a-3p			2								
	mmu-miR-125b-5p			4								
