# Supplementary material for: Adipose tissue from metabolic syndrome mice induces an aberrant miRNA signature highly relevant in prostate cancer development
Source: Mol Oncol. 2020 Sep 25;14(11):2868–83. doi: 10.1002/1878-0261.12788 (PMC7607170; doi:10.1002/1878-0261.12788)
Supplement: Supplementary file 6 — Table S6. Functional enrichment of the up modulated miRNAs. [file MOL2-14-2868-s006.pdf]

Table S6. Functional enrichment of target genes for up modulated miRNAs. DIANA-miRPath v3 tool

#	KEGG pathway	p-value	#miRNAs	#genes	genes							
1	Fatty acid metabolism (mmu01212)	5.49E-29	**4**	**14**	Scd2	Scd1	Ppt1	Pecr	Hadha	Fasn	Fads1	Elovl5
	mmu-miR-125a-3p			6	Echs1	Acsl1	Acox3	Acox1	Acat2	Acadsb		
	mmu-miR-214-3p			4								
	mmu-miR-101a-5p			3								
	mmu-miR-320-3p			6								
2	Fatty acid biosynthesis (mmu00061)	6.92E-17	**3**	**2**	Fasn	Acsl1						
	mmu-miR-125a-3p			1								
	mmu-miR-320-3p			2								
	mmu-miR-214-3p			1								
3	Fatty acid degradation (mmu00071)	6.26E-10	3	**12**	Aldh9a1	Hadha	Echs1	Cyp4a14	Aldh7a1	Aldh3a2	Aldh1b1	Acsl1
	mmu-miR-125a-3p			5	Acox3	Acox1	Acat2	Acadsb				
	mmu-miR-214-3p			5								
	mmu-miR-101a-5p			2								
4	Steroid biosynthesis (mmu00100)	6.62E-08	2	**6**	Sc5d	Sqle	Msmo1	Ebp	Dhcr24	Cyp51		
	mmu-miR-214-3p			4								
	mmu-miR-125a-3p			3								
5	Valine, leucine and isoleucine degradation (mmu00280)	1.73E-07	4	**15**	Hmgcs2	Oxct1	Mut	Hmgcs1	Hadha	Echs1	Bckdhb	Bckdha
	mmu-miR-214-3p			6	Aldh9a1	Aldh7a1	Aldh6a1	Aldh3a2	Aldh1b1	Acat2	Acadsb	
	mmu-miR-101a-5p			3								
	mmu-miR-125a-3p			3								
	mmu-miR-320-3p			6								
6	Fatty acid elongation (mmu00062)	1.62E-06	3	**4**	Hadha	Ppt1	Elovl5	Echs1				
	mmu-miR-214-3p			2								
	mmu-miR-125a-3p			2								
	mmu-miR-101a-5p			1								
7	N-Glycan biosynthesis (mmu00510)	6.15E-06	4	**11**	Alg1	Alg10b	Alg11	Alg2	Alg9	B4galt1	Ddost	Dpagt1
	mmu-miR-125a-3p			9	Ganab	Man1a2	Tusc3					
	mmu-miR-214-3p			3								
	mmu-miR-320-3p			2								
	mmu-miR-101a-5p			1								
8	Lysine degradation (mmu00310)	1.80E-05	5	**15**	Whsc1	Suv39h1	Nsd1	Kmt2c	Hadha	Echs1	Dlst	Aldh9a1
	mmu-miR-101a-5p			2	Aldh3a2	Aldh1b1	Acat2	Aass	Aldh7a1	Dot1l	Kmt2d	
	mmu-miR-125a-3p			5								
	mmu-miR-214-3p			7								
	mmu-miR-320-3p			4								
	mmu-miR-351-5p			1								
9	Protein processing in endoplasmic reticulum (mmu04141)	6.42E-05	8	**39**	Yod1	Wfs1	Ube2j1	Ube2g1	Tusc3	Ssr4	Ssr1	Sil1
	mmu-miR-125a-3p			17	Sec63	Sec61a1	Sec31a	Sec24c	Sec24b	Sec24a	Sec23b	Sar1a
	mmu-miR-214-3p			13	Rrbp1	Rbx1	Prkcsh	Preb	Nploc4	Nfe2l2	Mbtps2	Mapk8
	mmu-miR-17-3p			1	Man1a2	Lman2	Hsph1	Hsp90ab1	Hsp90aa1	Ganab	Erlec1	Eif2ak3
	mmu-miR-127-3p			2	Eif2ak1	Edem1	Dnaja2	Ddost	Atf6	Atf4	Amfr	
	mmu-miR-320-3p			6								
	mmu-miR-101a-5p			6								
	mmu-miR-205-5p			2								
	mmu-miR-351-5p			1								
10	Metabolic pathways (mmu01100)	0.000649362	5	**141**	Xdh	Uqcrfs1	Uqcrc1	Uox	Ugt2b5	Ugt2b36	Ugt2b34	Ugt1a7c
	mmu-miR-125a-3p			68	Ugt1a5	Ugt1a1	Tusc3	Thtpa	Tdo2	Tat	Synj2	St3gal2
	mmu-miR-214-3p			48	Sqle	Sptlc2	Spr	Sord	Sh3bp1	Sds	Scly	Sc5d
	mmu-miR-320-3p			46	Rrm2b	Rrm1	Rgn	Rdh11	Ptgs1	Ptgis	Prps1	Ppt1
	mmu-miR-101a-5p			14	Ppat	Pon1	Polr3g	Polr2b	Pnpo	Pkm	Pign	Pgm1
	mmu-miR-205-5p			2	Pfkl	Pemt	Pank2	Pah	Pafah1b1	Otc	Ndufa13	Mvd
					Mut	mt-Nd6	Msmo1	Mri1	Mgll	Me1	Mat2a	Mat1a
					Man1a2	Lpin2	Lipt2	Lipt1	Hsd3b7	Hprt	Hmgcs2	Hmgcs1
					Hao1	Hadha	H6pd	Grhpr	Gpi1	Gpam	Glul	Gldc
					Glb1	Ggt6	Gfpt1	Gclm	Gclc	Ganab	Ftcd	Fech
					Fbp1	Fasn	Etnk2	Etnk1	Echs1	Ebp	Dtymk	Dpyd
					Dpagt1	Dlst	Dhcr24	Dgat2	Ddost	Dak	Cyp51	Cyp4a14
					Cyp3a11	Cyp2c70	Cyp2a5	Cyp27a1	Cyp1a2	Cs	Cps1	Cox8a
					Chpf2	Chpf	Ces1d	Cers6	Cers5	Cds2	C1galt1	Bckdhb
					Bckdha	Baat	B4galt1	Atp6v1d	Atp5o	Atp5f1	Atp5a1	Alg9
					Alg2	Alg11	Alg10b	Alg1	Aldob	Aldh9a1	Aldh7a1	Aldh6a1
					Aldh3a2	Aldh1b1	Alas2	Akr1a1	Agmat	Agl	Adi1	Acsl1
					Acox3	Acox1	Acat2	Acadsb	Aass			
11	Biosynthesis of unsaturated fatty acids (mmu01040)	0.000842659	4	**9**	Scd2	Scd1	Pecr	Hadha	Fads1	Elovl5	Baat	Acox3
	mmu-miR-125a-3p			4	Acox1							
	mmu-miR-214-3p			2								
	mmu-miR-320-3p			4								
	mmu-miR-101a-5p			2								
12	Prostate cancer (mmu05215)	0.006275602	2	**5**	Pten	Pdpk1	Hsp90ab1	Hsp90aa1	Atf4			
	mmu-miR-205-5p											
	mmu-miR-127-3p											
13	PI3K-Akt signaling pathway (mmu04151)	0.006275602	2	**8**	Ywhae	Sgk1	Pten	Pdpk1	Mcl1	Hsp90ab1	Hsp90aa1	Atf4
	mmu-miR-205-5p											
	mmu-miR-674-5p											
	mmu-miR-127-3p											
