# Supplementary material for: Adipose tissue from metabolic syndrome mice induces an aberrant miRNA signature highly relevant in prostate cancer development
Source: Mol Oncol. 2020 Sep 25;14(11):2868–83. doi: 10.1002/1878-0261.12788 (PMC7607170; doi:10.1002/1878-0261.12788)
Supplement: Supplementary file 7 — Table S7. Lists of target genes associated to deregulated miRNAs. [file MOL2-14-2868-s007.pdf]

Table S7. Lists of target genes associated to up and down modulated miRNAs and those common to both groups

Target genes of up modulated miRNAs			
Up miRNAs			
Scd2	Sec31a	Pign	Atp5f1
Scd1	Sec24c	Pgm1	Atp5a1
Ppt1	Sec24b	Pfkl	Aldob
Pecr	Sec24a	Pemt	Alas2
Hadha	Sec23b	Pank2	Akr1a1
Fasn	Sar1a	Pah	Agmat
Fads1	Rrbp1	Pafah1b1	Agl
Elovl5	Rbx1	Otc	Adi1
Echs1	Prkcsh	Ndufa13	Ywhae
Acsl1	Preb	Mvd	Sgk1
Acox3	Nploc4	mt-Nd6	Pten
Acox1	Nfe2l2	Mri1	Pdpk1
Acat2	Mbtps2	Mgll	Mcl1
Acadsb	Mapk8	Me1	
Aldh9a1	Lman2	Mat2a	
Cyp4a14	Hsph1	Mat1a	
Aldh7a1	Hsp90ab1	Lpin2	
Aldh3a2	Hsp90aa1	Lipt2	
Aldh1b1	Erlec1	Lipt1	
Sc5d	Eif2ak3	Hsd3b7	
Sqle	Eif2ak1	Hprt	
Msmo1	Edem1	Hao1	
Ebp	Dnaja2	H6pd	
Dhcr24	Atf6	Grhpr	
Cyp51	Atf4	Gpi1	
Hmgcs2	Amfr	Gpam	
Oxct1	Xdh	Glul	
Mut	Uqcrfs1	Gldc	
Hmgcs1	Uqcrc1	Glb1	
Bckdhb	Uox	Ggt6	
Bckdha	Ugt2b5	Gfpt1	
Aldh6a1	Ugt2b36	Gclm	
Alg1	Ugt2b34	Gclc	
Alg10b	Ugt1a7c	Ftcd	
Alg11	Ugt1a5	Fech	
Alg2	Ugt1a1	Fbp1	
Alg9	Thtpa	Etnk2	
B4galt1	Tdo2	Etnk1	
Ddost	Tat	Dtymk	
Dpagt1	Synj2	Dpyd	
Ganab	St3gal2	Dgat2	
Man1a2	Sptlc2	Dak	
Tusc3	Spr	Cyp3a11	
Whsc1	Sord	Cyp2c70	
Suv39h1	Sh3bp1	Cyp2a5	
Nsd1	Sds	Cyp27a1	
Kmt2c	Scly	Cyp1a2	
Dlst	Rrm2b	Cs	
Aass	Rrm1	Cps1	
Dot1l	Rgn	Cox8a	
Kmt2d	Rdh11	Chpf2	
Yod1	Ptgs1	Chpf	
Wfs1	Ptgis	Ces1d	
Ube2j1	Prps1	Cers6	
Ube2g1	Ppat	Cers5	
Ssr4	Pon1	Cds2	
Ssr1	Polr3g	C1galt1	
Sil1	Polr2b	Baat	
Sec63	Pnpo	Atp6v1d	
Sec61a1	Pkm	Atp5o	

Target genes of down modulated miRNAs									
Down miRNAs									
Aacs	Auh	Cpt2	Elovl6	Igf1	Mcm5	Pgam1	Pvrl3	Slc7a5	Ube4b
Abat	Axin1	Crb2	Ep300	Igf1r	Mcm7	Pi4k2a	Rac1	Slco1c1	Ubqln1
Acaa2	B4galt1	Creb1	Epas1	Il6	Mdm2	Pi4k2b	Rac2	Smad1	Ubqln2
Acads	B4galt2	Creb3l2	Erbb2	Il7r	Med1	Pi4kb	Rad21	Smad2	Ubqln4
Acadsb	Bag2	Crebbp	Ern1	Impa2	Med12	Pik3c2a	Rad23b	Smad3	Uggt2
Acat1	Baiap2	Crk	Ero1l	Impad1	Med13	Pik3c2b	Rap1a	Smad4	Usp7
Acat2	Bak1	Csnk1d	Ero1lb	Inpp4a	Med13l	Pik3cb	Rap1b	Smad7	Vav2
Acox1	Bbc3	Csnk1e	Erp29	Inpp4b	Med14	Pik3cd	Rb1	Smc1a	Vcl
Acox3	Bcat1	Csnk2a1	Esr1	Inpp5a	Med17	Pik3r1	Rbl1	Smc3	Vegfa
Acsbg1	Bckdha	Ctgf	Ets1	Inpp5b	Med30	Pik3r2	Rbl2	Snai1	Vhl
Acsl1	Bcl2	Ctnnd1	Ezr	Inpp5d	Met	Pik3r3	Rcan2	Sod2	Vimp
Acsl3	Bcl2l11	Ctsl	Fads1	Inpp5e	Mgat2	Pikfyve	Rdx	Sos1	Vtn
Acsl6	Bcl6	Cul1	Fads2	Insr	Mgat3	Pip4k2a	Rela	Sos2	Wasf2
Actb	Bmp4	Cul2	Fasl	Ippk	Mgat4b	Pip4k2b	Ret	Src	Wasl
Actn2	Bmp7	Cyp4a10	Fasn	Iqgap1	Mgat5	Pip4k2c	Rnf5	Ssr1	Wee1
Actn4	Bmpr1a	Cyp4a12a	Fbxo25	Irs1	Mllt4	Pip5k1a	Rock1	Ssr3	Wfs1
Adh4	Bmpr2	Cyp4a14	Fbxo32	Itga5	Mob1a	Pip5k1c	Rock2	Ssr4	Whsc1
Adh5	Braf	Dbt	Fer	Itgav	Mob1b	Pkm	Rpn1	Ssx2ip	Whsc1l1
Agap2	Bub1	Ddx5	Fgfr1	Itgb1	Mpp5	Plaa	Rpn2	Stag1	Wnt2
Ajuba	Bub3	Derl1	Fgfr3	Itgb2	Mras	Plcb1	Rps6kb1	Stag2	Wnt5a
Akt1	Calm1	Dgka	Fh1	Itgb3	Mtm1	Plcd1	Rras	Stat1	Wwtr1
Aldh2	Calm2	Dgkd	Flnb	Itpk1	Mtor	Plce1	Rras2	Stat3	Xbp1
Aldh3a2	Calm3	Dgke	Fn1	Itpkb	Mut	Plk2	Rrbp1	Stk11	Yap1
Aldh6a1	Calr	Dgkh	Foxo1	Itpkc	Myc	Plk3	Rxra	Stk3	Yod1
Aldh7a1	Camk2a	Dio2	Foxo4	Itpr1	Ncoa1	Plod2	S1pr1	Stk4	Ywhab
Alg10b	Camk2d	Dld	Frs2	Itpr3	Ncoa2	Ppp1ca	S1pr4	Stt3a	Ywhae
Alg11	Camk2g	Dlg4	Fyn	Jun	Ncoa3	Ppp1cb	Sar1b	Stt3b	Ywhag
Alg14	Canx	Dlst	Fzd1	Kat2b	Ncor1	Ppp1r12a	Sav1	Stub1	Ywhah
Alg2	Capn2	Dnaja2	Fzd5	Kdr	Nf2	Ppp1r12b	Scd1	Suv39h1	Zbtb17
Alg5	Casp3	Dnajb1	Fzd7	Klf2	Nfe2l2	Ppp1r12c	Scd2	Suv420h1	
Alg6	Cblb	Dnajb12	G6pc	Kmt2b	Nfkb1	Ppp2ca	Sdc1	Svip	
Alg9	Ccna2	Dnajc10	Gab1	Kmt2c	Nfkbia	Ppp2cb	Sdc2	Synj1	
Amot	Ccnd1	Dnajc3	Gabarapl1	Kmt2d	Ngly1	Ppp2r1b	Sdc4	Synj2	
Anapc1	Ccnd2	Dolk	Ganab	Kras	Nlk	Ppp2r2a	Sec23a	Tbc1d4	
Anapc10	Ccne1	Dot1l	Gcdh	Lats1	Notch1	Ppt1	Sec23b	Tceb1	
Anapc2	Ccne2	Dpagt1	Gck	Lats2	Notch2	Preb	Sec24a	Tcf7	
Anapc4	Ccng2	Dvl1	Gls	Ldha	Nploc4	Prkaa1	Sec24b	Tcf7l1	
Anapc7	Cd44	Dvl3	Gpc1	Lef1	Nras	Prkaa2	Sec24c	Tcf7l2	
Ank2	Cdc14a	E2f1	Grb2	Llgl1	Nsd1	Prkab2	Sec24d	Tead1	
Ank3	Cdc14b	E2f2	Gsk3b	Lman2	Ogdh	Prkaca	Sec31a	Tfap4	
Aox3	Cdc16	E2f3	Hadh	Lmo7	Orc2	Prkacb	Sec61a1	Tfdp1	
Apc	Cdc23	E2f5	Hadha	Man1a	Orc4	Prkag2	Sec62	Tfdp2	
Ar	Cdc25a	Echs1	Hbegf	Man1a2	Os9	Prkca	Sec63	Tgfb1	
Araf	Cdc27	Edem1	Hdac2	Man1b1	Oxct1	Prkcb	Sel1l	Tgfb2	
Arhgef1	Cdc42	Edem3	Hdac3	Man1c1	Pak1	Prkcg	Serpine1	Tgfb3	
Arhgef12	Cdc7	Egfr	Hgf	Man2a1	Pak2	Prkci	Setd1a	Tgfbr1	
Arnt	Cdh1	Egln1	Hibadh	Man2a2	Pak4	Prkcsh	Setd1b	Tgfbr2	
Arnt2	Cdipt	Egln2	Hif1a	Map2k7	Pard6a	Prkcz	Setd2	Thra	
Atf4	Cdk2	Egln3	Hk1	Map3k5	Pcca	Prkdc	Setd7	Tiam1	
Atf6	Cdk4	Ehhadh	Hk2	Map3k7	Pck1	Prkx	Setd8	Timp3	
Atg12	Cdk7	Ehmt1	Hmgcs1	Mapk10	Pdgfb	Prmt1	Setdb1	Tjp1	
Atm	Cds2	Eif2ak1	Hmgcs2	Mapk14	Pdgfrb	Pten	Sgk1	Tlr4	
Atp1a1	Chek1	Eif2ak2	Homer1	Mapk8	Pdha1	Ptk2	Sil1	Tram1	
Atp1a2	Chek2	Eif2ak3	Hsd17b12	Mapk9	Pdhb	Ptpn1	Sirt3	Trp53bp2	
Atp1b1	Chuk	Eif2ak4	Hsp90aa1	March6	Pdia3	Ptpn11	Sirt6	Txndc5	
Atp1b2	Ckap4	Eif2s1	Hsp90ab1	Mbtps2	Pdia4	Ptprb	Skp2	Ube2d1	
Atp1b3	Col1a1	Eif4b	Hspa4l	Mcat	Pdia6	Ptprf	Slc16a10	Ube2d3	
Atp2a2	Col1a2	Elk1	Hspa5	Mcee	Pdk1	Ptprm	Slc16a2	Ube2g1	
Atr	Colgalt1	Elovl2	Id1	Mcm3	Pdpk1	Pvrl1	Slc1a5	Ube2g2	
Atxn3	Cpt1a	Elovl5	Id2	Mcm4	Pfkp	Pvrl2	Slc2a1	Ube2j1	

Target genes present only in up modulated miRNAs	
Pecr	Gfpt1
Aldh9a1	Gclm
Aldh1b1	Gclc
Sc5d	Ftcd
Sqle	Fech
Msmo1	Fbp1
Ebp	Etnk2
Dhcr24	Etnk1
Cyp51	Dtymk
Bckdhb	Dpyd
Alg1	Dgat2
Ddost	Dak
Tusc3	Cyp3a11
Aass	Cyp2c70
Sar1a	Cyp2a5
Rbx1	Cyp27a1
Hsph1	Cyp1a2
Erlec1	Cs
Amfr	Cps1
Xdh	Cox8a
Uqcrfs1	Chpf2
Uqcrc1	Chpf
Uox	Ces1d
Ugt2b5	Cers6
Ugt2b36	Cers5
Ugt2b34	C1galt1
Ugt1a7c	Baat
Ugt1a5	Atp6v1d
Ugt1a1	Atp5o
Thtpa	Atp5f1
Tdo2	Atp5a1
Tat	Aldob
St3gal2	Alas2
Sptlc2	Akr1a1
Spr	Agmat
Sord	Agl
Sh3bp1	Adi1
Sds	Mcl1
Scly	
Rrm2b	
Rrm1	
Rgn	
Rdh11	
Ptgs1	
Ptgis	
Prps1	
Ppat	
Pon1	
Polr3g	
Polr2b	
Pnpo	
Pign	
Pgm1	
Pfkl	
Pemt	
Pank2	
Pah	
Pafah1b1	
Otc	
Ndufa13	
Mvd	
mt-Nd6	
Mri1	
Mgll	
Me1	
Mat2a	
Mat1a	
Lpin2	
Lipt2	
Lipt1	
Hsd3b7	
Hprt	
Hao1	
H6pd	
Grhpr	
Gpi1	
Gpam	
Glul	
Gldc	
Glb1	
Ggt6	

Target genes common to up and down modulated miRNAs
Scd2
Scd1
Ppt1
Hadha
Fasn
Fads1
Elovl5
Echs1
Acsl1
Acox3
Acox1
Acat2
Acadsb
Cyp4a14
Aldh7a1
Aldh3a2
Hmgcs2
Oxct1
Mut
Hmgcs1
Bckdha
Aldh6a1
Alg10b
Alg11
Alg2
Alg9
B4galt1
Dpagt1
Ganab
Man1a2
Whsc1
Suv39h1
Nsd1
Kmt2c
Dlst
Dot1l
Kmt2d
Yod1
Wfs1
Ube2j1
Ube2g1
Ssr4
Ssr1
Sil1
Sec63
Sec61a1
Sec31a
Sec24c
Sec24b
Sec24a
Sec23b
Rrbp1
Prkcsh
Preb
Nploc4
Nfe2l2
Mbtps2
Mapk8
Lman2
Hsp90ab1
Hsp90aa1
Eif2ak3
Eif2ak1
Edem1
Dnaja2
Atf6
Atf4
Synj2
Pkm
Cds2
Ywhae
Sgk1
Pten
Pdpk1

Target genes present only in down modulated miRNAs						
Aacs	Cd44	Ets1	Man1a	Pikfyve	Setd2	Wnt2
Abat	Cdc14a	Ezr	Man1b1	Pip4k2a	Setd7	Wnt5a
Acaa2	Cdc14b	Fads2	Man1c1	Pip4k2b	Setd8	Wwtr1
Acads	Cdc16	Fasl	Man2a1	Pip4k2c	Setdb1	Xbp1
Acat1	Cdc23	Fbxo25	Man2a2	Pip5k1a	Sirt3	Yap1
Acsbg1	Cdc25a	Fbxo32	Map2k7	Pip5k1c	Sirt6	Ywhab
Acsl3	Cdc27	Fer	Map3k5	Plaa	Skp2	Ywhag
Acsl6	Cdc42	Fgfr1	Map3k7	Plcb1	Slc16a10	Ywhah
Actb	Cdc7	Fgfr3	Mapk10	Plcd1	Slc16a2	Zbtb17
Actn2	Cdh1	Fh1	Mapk14	Plce1	Slc1a5	
Actn4	Cdipt	Flnb	Mapk9	Plk2	Slc2a1	
Adh4	Cdk2	Fn1	March6	Plk3	Slc7a5	
Adh5	Cdk4	Foxo1	Mcat	Plod2	Slco1c1	
Agap2	Cdk7	Foxo4	Mcee	Ppp1ca	Smad1	
Ajuba	Chek1	Frs2	Mcm3	Ppp1cb	Smad2	
Akt1	Chek2	Fyn	Mcm4	Ppp1r12a	Smad3	
Aldh2	Chuk	Fzd1	Mcm5	Ppp1r12b	Smad4	
Alg14	Ckap4	Fzd5	Mcm7	Ppp1r12c	Smad7	
Alg5	Col1a1	Fzd7	Mdm2	Ppp2ca	Smc1a	
Alg6	Col1a2	G6pc	Med1	Ppp2cb	Smc3	
Amot	Colgalt1	Gab1	Med12	Ppp2r1b	Snai1	
Anapc1	Cpt1a	Gabarapl1	Med13	Ppp2r2a	Sod2	
Anapc10	Cpt2	Gcdh	Med13l	Prkaa1	Sos1	
Anapc2	Crb2	Gck	Med14	Prkaa2	Sos2	
Anapc4	Creb1	Gls	Med17	Prkab2	Src	
Anapc7	Creb3l2	Gpc1	Med30	Prkaca	Ssr3	
Ank2	Crebbp	Grb2	Met	Prkacb	Ssx2ip	
Ank3	Crk	Gsk3b	Mgat2	Prkag2	Stag1	
Aox3	Csnk1d	Hadh	Mgat3	Prkca	Stag2	
Apc	Csnk1e	Hbegf	Mgat4b	Prkcb	Stat1	
Ar	Csnk2a1	Hdac2	Mgat5	Prkcg	Stat3	
Araf	Ctgf	Hdac3	Mllt4	Prkci	Stk11	
Arhgef1	Ctnnd1	Hgf	Mob1a	Prkcz	Stk3	
Arhgef12	Ctsl	Hibadh	Mob1b	Prkdc	Stk4	
Arnt	Cul1	Hif1a	Mpp5	Prkx	Stt3a	
Arnt2	Cul2	Hk1	Mras	Prmt1	Stt3b	
Atg12	Cyp4a10	Hk2	Mtm1	Ptk2	Stub1	
Atm	Cyp4a12a	Homer1	Mtor	Ptpn1	Suv420h1	
Atp1a1	Dbt	Hsd17b12	Myc	Ptpn11	Svip	
Atp1a2	Ddx5	Hspa4l	Ncoa1	Ptprb	Synj1	
Atp1b1	Derl1	Hspa5	Ncoa2	Ptprf	Tbc1d4	
Atp1b2	Dgka	Id1	Ncoa3	Ptprm	Tceb1	
Atp1b3	Dgkd	Id2	Ncor1	Pvrl1	Tcf7	
Atp2a2	Dgke	Igf1	Nf2	Pvrl2	Tcf7l1	
Atr	Dgkh	Igf1r	Nfkb1	Pvrl3	Tcf7l2	
Atxn3	Dio2	Il6	Nfkbia	Rac1	Tead1	
Auh	Dld	Il7r	Ngly1	Rac2	Tfap4	
Axin1	Dlg4	Impa2	Nlk	Rad21	Tfdp1	
B4galt2	Dnajb1	Impad1	Notch1	Rad23b	Tfdp2	
Bag2	Dnajb12	Inpp4a	Notch2	Rap1a	Tgfb1	
Baiap2	Dnajc10	Inpp4b	Nras	Rap1b	Tgfb2	
Bak1	Dnajc3	Inpp5a	Ogdh	Rb1	Tgfb3	
Bbc3	Dolk	Inpp5b	Orc2	Rbl1	Tgfbr1	
Bcat1	Dvl1	Inpp5d	Orc4	Rbl2	Tgfbr2	
Bcl2	Dvl3	Inpp5e	Os9	Rcan2	Thra	
Bcl2l11	E2f1	Insr	Pak1	Rdx	Tiam1	
Bcl6	E2f2	Ippk	Pak2	Rela	Timp3	
Bmp4	E2f3	Iqgap1	Pak4	Ret	Tjp1	
Bmp7	E2f5	Irs1	Pard6a	Rnf5	Tlr4	
Bmpr1a	Edem3	Itga5	Pcca	Rock1	Tram1	
Bmpr2	Egfr	Itgav	Pck1	Rock2	Trp53bp2	
Braf	Egln1	Itgb1	Pdgfb	Rpn1	Txndc5	
Bub1	Egln2	Itgb2	Pdgfrb	Rpn2	Ube2d1	
Bub3	Egln3	Itgb3	Pdha1	Rps6kb1	Ube2d3	
Calm1	Ehhadh	Itpk1	Pdhb	Rras	Ube2g2	
Calm2	Ehmt1	Itpkb	Pdia3	Rras2	Ube4b	
Calm3	Eif2ak2	Itpkc	Pdia4	Rxra	Ubqln1	
Calr	Eif2ak4	Itpr1	Pdia6	S1pr1	Ubqln2	
Camk2a	Eif2s1	Itpr3	Pdk1	S1pr4	Ubqln4	
Camk2d	Eif4b	Jun	Pfkp	Sar1b	Uggt2	
Camk2g	Elk1	Kat2b	Pgam1	Sav1	Usp7	
Canx	Elovl2	Kdr	Pi4k2a	Sdc1	Vav2	
Capn2	Elovl6	Klf2	Pi4k2b	Sdc2	Vcl	
Casp3	Ep300	Kmt2b	Pi4kb	Sdc4	Vegfa	
Cblb	Epas1	Kras	Pik3c2a	Sec23a	Vhl	
Ccna2	Erbb2	Lats1	Pik3c2b	Sec24d	Vimp	
Ccnd1	Ern1	Lats2	Pik3cb	Sec62	Vtn	
Ccnd2	Ero1l	Ldha	Pik3cd	Sel1l	Wasf2	
Ccne1	Ero1lb	Lef1	Pik3r1	Serpine1	Wasl	
Ccne2	Erp29	Llgl1	Pik3r2	Setd1a	Wee1	
Ccng2	Esr1	Lmo7	Pik3r3	Setd1b	Whsc1l1	
