# Supplementary material for: Adipose tissue from metabolic syndrome mice induces an aberrant miRNA signature highly relevant in prostate cancer development
Source: Mol Oncol. 2020 Sep 25;14(11):2868–83. doi: 10.1002/1878-0261.12788 (PMC7607170; doi:10.1002/1878-0261.12788)
Supplement: Supplementary file 8 — Table S8. Functional enrichment of target genes present only in down modulated miRNAs. [file MOL2-14-2868-s008.pdf]

Table S8. Functional enrichment of target genes present only in down modulated miRNAs. ClueGo

Function	Groups	Group Genes															
Fatty acid degradation	Group00	Acaa2	Acads	Acat1	Acsbg1	Acsl3	Acsl6	Adh4	Adh5	Aldh2	Cpt1a	Cpt2	Cyp4a10	Cyp4a12a	Ehhadh	Gcdh	Hadh
Valine, leucine and isoleucine degradation	Group01	Aacs	Abat	Acaa2	Acads	Acat1	Aldh2	Aox3	Auh	Bcat1	Dbt	Dld	Ehhadh	Hadh	Hibadh	Mcee	Pcca
Protein processing in endoplasmic reticulum	Group02	Atxn3	Bag2	Bak1	Bcl2	Calr	Canx	Capn2	Ckap4	Cul1	Derl1	Dnajb1	Dnajb12	Dnajc10	Dnajc3	Edem3	Eif2ak2
		Eif2ak4	Eif2s1	Ern1	Ero1l	Ero1lb	Erp29	Hspa4l	Hspa5	Man1a	Man1b1	Man1c1	Map2k7	Map3k5	Mapk10	Mapk9	March6
		Ngly1	Os9	Pdia3	Pdia4	Pdia6	Plaa	Rad23b	Rnf5	Rpn1	Rpn2	Sar1b	Sec23a	Sec24d	Sec62	Sel1l	Selenos
		Ssr3	Stt3a	Stt3b	Stub1	Svip	Tram1	Txndc5	Ube2d1	Ube2d3	Ube2g2	Ube4b	Ubqln1	Ubqln2	Ubqln4	Uggt2	Xbp1
Lysine degradation	Group03	Acat1	Aldh2	Colgalt1	Ehhadh	Ehmt1	Gcdh	Hadh	Kmt2b	Kmt5a	Kmt5b	Nsd3	Ogdh	Plod2	Setd1a	Setd1b	Setd2
		Setd7	Setdb1														
TGF-beta signaling pathway	Group04	Bmp4	Bmp7	Bmpr1a	Bmpr2	Crebbp	Cul1	E2f5	Ep300	Id1	Id2	Myc	Ppp2ca	Ppp2cb	Ppp2r1b	Rbl1	Rock1
		Rps6kb1	Smad1	Smad2	Smad3	Smad4	Smad7	Tfdp1	Tgfb1	Tgfb2	Tgfb3	Tgfbr1	Tgfbr2				
N-Glycan biosynthesis	Group05	Alg14	Alg5	Alg6	B4galt2	Dolk	Man1a	Man1b1	Man1c1	Man2a1	Man2a2	Mgat2	Mgat3	Mgat4b	Mgat5	Rpn1	Rpn2
		Stt3a	Stt3b														
Hippo signaling pathway	Group06|Group07	Actb	Ajuba	Amot	Apc	Axin1	Bbc3	Bmp4	Bmp7	Bmpr1a	Bmpr2	Ccn2	Ccnd1	Ccnd2	Cdh1	Crb2	Csnk1d
		Csnk1e	Dlg4	Dvl1	Dvl3	Fzd1	Fzd5	Fzd7	Gsk3b	Id1	Id2	Itgb2	Lats1	Lats2	Lef1	Llgl1	Mob1a
		Mob1b	Mpp5	Myc	Nf2	Pak1	Pard6a	Ppp1ca	Ppp1cb	Ppp2ca	Ppp2cb	Ppp2r1b	Ppp2r2a	Prkci	Prkcz	Sav1	Serpine1
		Smad1	Smad2	Smad3	Smad4	Smad7	Stk3	Tcf7	Tcf7l1	Tcf7l2	Tead1	Tgfb1	Tgfb2	Tgfb3	Tgfbr1	Tgfbr2	Trp53bp2
		Wnt2	Wnt5a	Wwtr1	Yap1	Ywhab	Ywhag	Ywhah									
Adherens junction	Group08	Actb	Actn4	Afdn	Baiap2	Cdc42	Cdh1	Crebbp	Csnk2a1	Ctnnd1	Egfr	Ep300	Erbb2	Fer	Fgfr1	Fyn	Igf1r
		Insr	Iqgap1	Lef1	Lmo7	Map3k7	Met	Nectin1	Nectin2	Nectin3	Nlk	Ptpn1	Ptprb	Ptprf	Ptprm	Rac1	Rac2
		Smad2	Smad3	Smad4	Snai1	Src	Ssx2ip	Tcf7	Tcf7l1	Tcf7l2	Tgfbr1	Tgfbr2	Tjp1	Vcl	Wasf2	Wasl	
Tight junction	Group09	Actb	Actn4	Afdn	Amot	Ccnd1	Cdc42	Cdk4	Erbb2	Ezr	Itgb1	Jun	Llgl1	Map2k7	Map3k5	Mapk10	Mapk9
		Mpp5	Nf2	Pard6a	Ppp2ca	Ppp2cb	Ppp2r1b	Ppp2r2a	Prkaa1	Prkaa2	Prkab2	Prkaca	Prkacb	Prkag2	Prkci	Prkcz	Rac1
		Rap1a	Rdx	Rock1	Rock2	Src	Stk11	Tiam1	Tjp1	Wasl							
Thyroid hormone signaling pathway	Group10	Actb	Akt1	Atp1a1	Atp1a2	Atp1b1	Atp1b2	Atp1b3	Atp2a2	Bmp4	Ccnd1	Crebbp	Dio2	Ep300	Esr1	Foxo1	Gsk3b
		Hdac2	Hdac3	Hif1a	Itgav	Itgb3	Kat2b	Kras	Mdm2	Med1	Med12	Med13	Med13l	Med14	Med17	Med30	Mtor
		Myc	Ncoa1	Ncoa2	Ncoa3	Ncor1	Notch1	Notch2	Nras	Pfkp	Pik3cb	Pik3cd	Pik3r1	Pik3r2	Pik3r3	Plcb1	Plcd1
		Plce1	Prkaca	Prkacb	Prkca	Prkcb	Prkcg	Rcan2	Rxra	Slc16a10	Slc16a2	Slc2a1	Slco1c1	Src	Stat1	Tbc1d4	Thra
Central carbon metabolism in cancer	Group11	Akt1	Egfr	Erbb2	Fgfr1	Fgfr3	Gck	Gls	Hif1a	Hk1	Hk2	Kras	Ldha	Met	Mtor	Myc	Nras
		Pdgfrb	Pdha1	Pdhb	Pdk1	Pfkp	Pgam1	Pik3cb	Pik3cd	Pik3r1	Pik3r2	Pik3r3	Ret	Sirt3	Sirt6	Slc1a5	Slc2a1
		Slc7a5															
MicroRNAs in cancer	Group12	Apc	Atm	Bak1	Bcl2	Bcl2l11	Bmpr2	Casp3	Ccnd1	Ccnd2	Ccne1	Ccne2	Cd44	Cdc25a	Crebbp	Crk	E2f1
		E2f2	E2f3	Egfr	Ep300	Erbb2	Ezr	Fgfr3	Gls	Grb2	Irs1	Itga5	Itgb3	Kras	Mdm2	Met	Mtor
		Myc	Nfkb1	Notch1	Notch2	Nras	Pak4	Pdgfb	Pdgfrb	Prkca	Prkcb	Prkcg	Rdx	Rock1	Sos1	Sos2	Stat3
		Tgfb2	Timp3	Vegfa													
HIF-1 signaling pathway	Group13	Akt1	Arnt	Bcl2	Camk2a	Camk2d	Camk2g	Crebbp	Cul2	Egfr	Egln1	Egln2	Egln3	Eloc	Ep300	Erbb2	Hif1a
		Hk1	Hk2	Igf1	Igf1r	Il6	Insr	Ldha	Mtor	Nfkb1	Pdha1	Pdhb	Pdk1	Pik3cb	Pik3cd	Pik3r1	Pik3r2
		Pik3r3	Prkca	Prkcb	Prkcg	Rela	Rps6kb1	Serpine1	Slc2a1	Stat3	Tlr4	Vegfa	Vhl				
FoxO signaling pathway	Group14	Agap2	Akt1	Araf	Atg12	Atm	Bcl2l11	Bcl6	Braf	Ccnd1	Ccnd2	Ccng2	Cdk2	Chuk	Crebbp	Csnk1e	Egfr
		Ep300	Fasl	Fbxo25	Fbxo32	Foxo1	Foxo4	G6pc	Gabarapl1	Grb2	Homer1	Igf1	Igf1r	Il6	Il7r	Insr	Irs1
		Klf2	Kras	Mapk10	Mapk14	Mapk9	Mdm2	Nlk	Nras	Pck1	Pik3cb	Pik3cd	Pik3r1	Pik3r2	Pik3r3	Plk2	Plk3
		Prkaa1	Prkaa2	Prkab2	Prkag2	Prmt1	Rbl2	S1pr1	S1pr4	Setd7	Skp2	Smad2	Smad3	Smad4	Sod2	Sos1	Sos2
		Stat3	Stk11	Stk4	Tgfb1	Tgfb2	Tgfb3	Tgfbr1	Tgfbr2	Usp7							
Phosphatidylinositol signaling system	Group15	Calm1	Calm2	Calm3	Cdipt	Dgka	Dgkd	Dgke	Dgkh	Impa2	Impad1	Inpp4a	Inpp4b	Inpp5a	Inpp5b	Inpp5d	Inpp5e
		Ippk	Itpk1	Itpkb	Itpkc	Itpr1	Itpr3	Mtm1	Pi4k2a	Pi4k2b	Pi4kb	Pik3c2a	Pik3c2b	Pik3cb	Pik3cd	Pik3r1	Pik3r2
		Pik3r3	Pikfyve	Pip4k2a	Pip4k2b	Pip4k2c	Pip5k1a	Pip5k1c	Plcb1	Plcd1	Plce1	Prkca	Prkcb	Prkcg	Synj1		
Human T-cell leukemia virus 1 infection	Group16	Akt1	Anapc1	Anapc10	Anapc2	Anapc4	Anapc7	Araf	Atm	Atr	Braf	Bub1	Bub3	Calr	Canx	Ccna2	Ccnd1
		Ccnd2	Ccne1	Ccne2	Cdc16	Cdc23	Cdc25a	Cdc27	Cdk2	Cdk4	Chek1	Chek2	Chuk	Creb1	Creb3l2	Crebbp	E2f1
		E2f2	E2f3	Elk1	Ep300	Ets1	Foxo1	Hdac2	Igf1	Igf1r	Il6	Insr	Irs1	Itgb2	Jun	Kat2b	Kras
		Mapk10	Mapk14	Mapk9	Mtor	Myc	Nfkb1	Nfkbia	Nras	Pik3cb	Pik3cd	Pik3r1	Pik3r2	Pik3r3	Prkaa1	Prkaa2	Prkab2
		Prkaca	Prkacb	Prkag2	Rb1	Rela	Rps6kb1	Slc2a1	Smad2	Smad3	Smad4	Sod2	Tgfb1	Tgfb2	Tgfb3	Tgfbr1	Tgfbr2
Autophagy	Group17	Actb	Akt1	Atg12	Atm	Bak1	Bbc3	Bcl2	Bcl2l11	Capn2	Casp3	Ccna2	Ccnd1	Chuk	Cpt1a	Creb1	Creb3l2
		Ctsl	Ehmt1	Eif2ak4	Eif2s1	Ern1	Fasl	Foxo1	G6pc	Gabarapl1	Hdac2	Hif1a	Igf1	Igf1r	Insr	Irs1	Itpr1
		Itpr3	Jun	Kras	Map3k5	Map3k7	Mapk10	Mapk9	Mras	Mtor	Nfkb1	Nfkbia	Nras	Pck1	Pfkp	Pik3cb	Pik3cd
		Pik3r1	Pik3r2	Pik3r3	Ppp2ca	Ppp2cb	Ppp2r1b	Ppp2r2a	Prkaa1	Prkaa2	Prkab2	Prkaca	Prkacb	Prkag2	Rela	Rps6kb1	Rras
		Rras2	Sod2	Stk11													
Focal adhesion	Group18	Actb	Actn4	Afdn	Akt1	Araf	Bcl2	Braf	Calm1	Calm2	Calm3	Capn2	Casp3	Ccna2	Ccnd1	Ccnd2	Ccne1
		Ccne2	Cdc42	Cdh1	Cdk2	Chuk	Col1a1	Col1a2	Creb1	Creb3l2	Crebbp	Crk	Ctnnd1	E2f1	E2f2	E2f3	Egfr
		Elk1	Ep300	Erbb2	Ets1	Fasl	Fgfr1	Fgfr3	Flnb	Fn1	Foxo4	Fyn	Gab1	Grb2	Gsk3b	Hgf	Id1
		Igf1	Igf1r	Il6	Insr	Itga5	Itgav	Itgb1	Itgb2	Itgb3	Jun	Kdr	Kras	Map2k7	Map3k5	Map3k7	Mapk10
		Mapk14	Mapk9	Met	Mras	Myc	Nfkb1	Nfkbia	Nlk	Nras	Pak1	Pak2	Pak4	Pard6a	Pdgfb	Pdgfrb	Pik3cb
		Pik3cd	Pik3r1	Pik3r2	Pik3r3	Pip5k1c	Plcb1	Plce1	Ppp1ca	Ppp1cb	Ppp1r12a	Ppp1r12b	Ppp1r12c	Prkaca	Prkacb	Prkca	Prkcb
		Prkcg	Prkci	Prkcz	Ptk2	Ptpn11	Rac1	Rac2	Rap1a	Rap1b	Rb1	Rela	Rock1	Rock2	Rras	Rras2	Smad2
		Smad3	Smad4	Sos1	Sos2	Src	Stat1	Stat3	Stk3	Stk4	Tgfb1	Tgfb2	Tgfb3	Tgfbr1	Tgfbr2	Tiam1	Tlr4
		Vav2	Vcl	Vegfa	Vtn	Ywhab											
AGE-RAGE signaling pathway in diabetic complications	Group19	Actb	Actn4	Afdn	Akt1	Bcl2	Calr	Casp3	Ccnd1	Cdc42	Cdk4	Chuk	Col1a1	Col1a2	Creb1	Creb3l2	Ctnnd1
		Egfr	Ezr	Fasl	Fn1	Foxo1	Grb2	Il6	Itgb1	Itgb2	Jun	Kras	Map2k7	Mapk10	Mapk14	Mapk9	Nfkb1
		Nfkbia	Nras	Pik3cb	Pik3cd	Pik3r1	Pik3r2	Pik3r3	Plcb1	Plcd1	Plce1	Ppp2ca	Ppp2cb	Ppp2r1b	Ppp2r2a	Prkaca	Prkacb
		Prkca	Prkcb	Prkcg	Prkcz	Ptk2	Ptpn11	Rac1	Rac2	Rap1a	Rap1b	Rela	Rock1	Rock2	Serpine1	Smad2	Smad3
		Smad4	Sos1	Sos2	Src	Stat1	Stat3	Tgfb1	Tgfb2	Tgfb3	Tgfbr1	Tgfbr2	Tlr4	Vav2	Vcl	Vegfa	
Prostate cancer	Group20	Actb	Actn4	Akt1	Apc	Ar	Araf	Atm	Atr	Axin1	Bak1	Bcl2	Bcl2l11	Braf	Capn2	Casp3	Ccna2
		Ccnd1	Ccnd2	Ccne1	Ccne2	Cdc42	Cdk2	Cdk4	Chuk	Col1a1	Col1a2	Cpt1a	Creb1	Creb3l2	Crebbp	Crk	Dvl1
		Dvl3	E2f1	E2f2	E2f3	Egfr	Eif2ak2	Eif2ak4	Eif2s1	Eif4b	Elk1	Ep300	Erbb2	Fasl	Fgfr1	Fgfr3	Flnb
		Fn1	Foxo1	Fyn	Fzd1	Fzd5	Fzd7	G6pc	Grb2	Gsk3b	Hdac2	Hgf	Igf1	Igf1r	Il6	Il7r	Insr
		Irs1	Itga5	Itgav	Itgb1	Itgb3	Jun	Kdr	Kras	Lef1	Llgl1	Map3k7	Mapk10	Mapk9	Mdm2	Met	Mpp5
		Mtor	Myc	Nfkb1	Nfkbia	Notch1	Notch2	Nras	Pak1	Pak2	Pak4	Pard6a	Pck1	Pdgfb	Pdgfrb	Pfkp	Pik3cb
		Pik3cd	Pik3r1	Pik3r2	Pik3r3	Pip5k1c	Ppp1ca	Ppp1cb	Ppp1r12a	Ppp1r12b	Ppp1r12c	Ppp2ca	Ppp2cb	Ppp2r1b	Ppp2r2a	Prkaa1	Prkaa2
		Prkab2	Prkaca	Prkacb	Prkag2	Prkca	Prkcb	Prkcg	Prkci	Prkcz	Ptk2	Rac1	Rac2	Rap1a	Rap1b	Rb1	Rbl1
		Rbl2	Rela	Rock1	Rock2	Rps6kb1	Rxra	Sos1	Sos2	Src	Stat1	Stat3	Stk11	Tcf7	Tcf7l1	Tcf7l2	Tlr4
		Vav2	Vcl	Vegfa	Vtn	Wnt2	Wnt5a	Ywhab	Ywhag	Ywhah							
Gastric cancer	Group21	Actb	Akt1	Apc	Araf	Arnt	Axin1	Bak1	Bcl2	Braf	Calm1	Calm2	Calm3	Camk2a	Camk2d	Camk2g	Ccnd1
		Ccnd2	Ccne1	Ccne2	Cdh1	Cdk2	Cdk4	Creb1	Creb3l2	Crebbp	Csnk1e	Csnk2a1	Cul1	Dvl1	Dvl3	E2f1	E2f2
		E2f3	Egfr	Elk1	Ep300	Erbb2	Esr1	Fgfr1	Fh1	Fzd1	Fzd5	Fzd7	Gab1	Grb2	Gsk3b	Hgf	Igf1
		Igf1r	Itpr1	Itpr3	Jun	Kras	Lef1	Map3k7	Mapk10	Mapk9	Met	Mtor	Myc	Ncoa1	Ncoa3	Nlk	Notch1
		Notch2	Nras	Pik3cb	Pik3cd	Pik3r1	Pik3r2	Pik3r3	Plcb1	Prkaca	Prkacb	Prkca	Prkcb	Prkcg	Rac1	Rac2	Rap1a
		Rap1b	Rb1	Rock2	Rps6kb1	Rxra	Smad2	Smad3	Smad4	Sos1	Sos2	Tcf7	Tcf7l1	Tcf7l2	Tgfb1	Tgfb2	Tgfb3
		Tgfbr1	Tgfbr2	Wnt2	Wnt5a												
Longevity regulating pathway	Group22	Akt1	Araf	Atp1a1	Atp1a2	Atp1b1	Atp1b2	Atp1b3	Braf	Casp3	Cdc42	Chuk	Dvl1	Dvl3	Eif4b	Fasl	Foxo1
		Fyn	Fzd1	Fzd5	Fzd7	G6pc	Grb2	Gsk3b	Hdac2	Hk1	Hk2	Igf1	Igf1r	Insr	Irs1	Itgb2	Kdr
		Kras	Mapk14	Mtor	Nras	Pak1	Pik3cb	Pik3cd	Pik3r1	Pik3r2	Pik3r3	Prkaa1	Prkaa2	Prkab2	Prkaca	Prkacb	Prkag2
		Prkca	Prkcb	Prkcg	Ptk2	Ptpn11	Rac1	Rac2	Rps6kb1	Skp2	Slc7a5	Sod2	Sos1	Sos2	Src	Stk11	Vav2
		Vegfa	Wnt2	Wnt5a													
Ras signaling pathway	Group23	Afdn	Akt1	Arhgef12	Atp1a1	Atp1a2	Atp1b1	Atp1b2	Atp1b3	Atp2a2	Bcl2	Braf	Calm1	Calm2	Calm3	Camk2a	Camk2d
		Camk2g	Cblb	Cdc42	Chuk	Creb1	Creb3l2	Crebbp	Crk	Cyp4a10	Cyp4a12a	Egfr	Elk1	Ep300	Ets1	Fasl	Fgfr1
		Fgfr3	Foxo4	Frs2	Gab1	Grb2	Gsk3b	Hgf	Igf1	Igf1r	Il6	Insr	Irs1	Itpr1	Itpr3	Jun	Kdr
		Kras	Map2k7	Map3k5	Mapk10	Mapk14	Mapk9	Mdm2	Met	Mras	Nfkb1	Nfkbia	Nras	Pak1	Pak2	Pak4	Pdgfb
		Pdgfrb	Pik3cb	Pik3cd	Pik3r1	Pik3r2	Pik3r3	Plcb1	Plce1	Plk3	Ppp1ca	Ppp1cb	Ppp1r12a	Prkaca	Prkacb	Prkca	Prkcb
		Prkcg	Prkcz	Ptk2	Ptpn11	Rac1	Rac2	Rap1a	Rap1b	Rela	Rock1	Rock2	Rras	Rras2	Sos1	Sos2	Src
		Stat1	Stat3	Stk4	Tiam1	Vav2	Vegfa	Wasl									
Neurotrophin signaling pathway	Group24	Actb	Akt1	Atm	Bak1	Bbc3	Bcl2	Bcl2l11	Braf	Calm1	Calm2	Calm3	Calr	Camk2a	Camk2d	Camk2g	Capn2
		Casp3	Cdc42	Chuk	Cpt1a	Creb1	Creb3l2	Crk	Ctsl	Ehmt1	Eif2s1	Ern1	Fasl	Foxo1	Frs2	Fyn	G6pc
		Gab1	Grb2	Gsk3b	Hdac2	Igf1	Igf1r	Il6	Insr	Irs1	Itgb3	Itpr1	Itpr3	Jun	Kras	Map2k7	Map3k5
		Map3k7	Mapk10	Mapk14	Mapk9	Mtor	Nfkb1	Nfkbia	Nras	Pck1	Pik3cb	Pik3cd	Pik3r1	Pik3r2	Pik3r3	Plcb1	Ppp1ca
		Ppp1cb	Ppp2ca	Ppp2cb	Ppp2r1b	Ppp2r2a	Prkaa1	Prkaa2	Prkab2	Prkaca	Prkacb	Prkag2	Prkcb	Prkcz	Ptpn1	Ptpn11	Ptprf
		Rac1	Rap1a	Rap1b	Rela	Rps6kb1	Rxra	Serpine1	Slc2a1	Smad2	Smad3	Sod2	Sos1	Sos2	Stat1	Stat3	Stk11
		Tbc1d4	Tgfb1	Tgfb2	Tgfb3	Tgfbr1	Tgfbr2	Tlr4	Xbp1								
Pathways in cancer	Group25	Actn4	Akt1	Apc	Ar	Araf	Arhgef1	Arhgef12	Arnt	Arnt2	Atm	Atr	Axin1	Bak1	Bbc3	Bcl2	Bcl2l11
		Bmp4	Braf	Calm1	Calm2	Calm3	Camk2a	Camk2d	Camk2g	Casp3	Ccna2	Ccnd1	Ccnd2	Ccne1	Ccne2	Cdc42	Cdh1
		Cdk2	Cdk4	Chek1	Chuk	Col1a1	Col1a2	Creb1	Creb3l2	Crebbp	Crk	Cul1	Cul2	Dvl1	Dvl3	E2f1	E2f2
		E2f3	Egfr	Egln1	Egln2	Egln3	Eif2ak2	Eif2ak4	Eif2s1	Eif4b	Elk1	Eloc	Ep300	Epas1	Erbb2	Esr1	Ets1
		Fasl	Fgfr1	Fgfr3	Fh1	Fn1	Foxo1	Fzd1	Fzd5	Fzd7	G6pc	Gab1	Grb2	Gsk3b	Hdac2	Hdac3	Hgf
		Hif1a	Igf1	Igf1r	Il6	Il7r	Insr	Irs1	Itga5	Itgav	Itgb1	Itgb3	Jun	Kat2b	Kdr	Kras	Lef1
		Llgl1	Mapk10	Mapk9	Mdm2	Met	Mpp5	Mtor	Myc	Ncoa1	Ncoa3	Nfkb1	Nfkbia	Notch1	Notch2	Nras	Pard6a
		Pck1	Pdgfb	Pdgfrb	Pik3cb	Pik3cd	Pik3r1	Pik3r2	Pik3r3	Plcb1	Ppp2ca	Ppp2cb	Ppp2r1b	Ppp2r2a	Prkaa1	Prkaa2	Prkaca
		Prkacb	Prkca	Prkcb	Prkcg	Prkci	Prkcz	Ptk2	Rac1	Rac2	Rb1	Rbl1	Rbl2	Rela	Ret	Rock1	Rock2
		Rps6kb1	Rxra	Skp2	Slc2a1	Smad2	Smad3	Smad4	Sos1	Sos2	Src	Stat1	Stat3	Stk11	Stk4	Tcf7	Tcf7l1
		Tcf7l2	Tgfb1	Tgfb2	Tgfb3	Tgfbr1	Tgfbr2	Tlr4	Usp7	Vegfa	Vhl	Vtn	Wnt2	Wnt5a	Ywhab	Ywhag	Ywhah
		Zbtb17															

Glioma	Group26	Actb	Actn4	Afdn	Akt1	Araf	Bak1	Bcl2	Braf	Calm1	Calm2	Calm3	Camk2a	Camk2d	Camk2g	Capn2	Casp3
		Ccnd1	Ccnd2	Cdc42	Cdh1	Cdk4	Chuk	Col1a1	Col1a2	Crk	Ctnnd1	E2f1	E2f2	E2f3	Egfr	Elk1	Erbb2
		Ets1	Ezr	Fasl	Fgfr1	Fgfr3	Flnb	Fn1	Foxo4	Fyn	Gab1	Grb2	Gsk3b	Hgf	Id1	Igf1	Igf1r
		Insr	Itga5	Itgav	Itgb1	Itgb2	Itgb3	Jun	Kdr	Kras	Map2k7	Map3k5	Map3k7	Mapk10	Mapk14	Mapk9	Mdm2
		Met	Mras	Mtor	Myc	Nfkb1	Nlk	Nras	Pak1	Pak2	Pak4	Pard6a	Pdgfb	Pdgfrb	Pik3cb	Pik3cd	Pik3r1
		Pik3r2	Pik3r3	Pip5k1c	Plcb1	Plce1	Ppp1ca	Ppp1cb	Ppp1r12a	Ppp1r12b	Ppp1r12c	Prkaca	Prkacb	Prkca	Prkcb	Prkcg	Prkci
		Prkcz	Ptk2	Ptpn11	Rac1	Rac2	Rap1a	Rap1b	Rb1	Rela	Rock1	Rock2	Rras	Rras2	Sos1	Sos2	Src
		Stk3	Stk4	Tgfb1	Tgfb2	Tgfb3	Tgfbr1	Tgfbr2	Tiam1	Vav2	Vcl	Vegfa	Vtn				
Hepatitis B	Group27	Actn4	Akt1	Anapc1	Anapc10	Anapc2	Anapc4	Anapc7	Apc	Araf	Atm	Atr	Axin1	Bak1	Bcl2	Bcl2l11	Braf
		Bub3	Calm1	Calm2	Calm3	Calr	Canx	Casp3	Ccna2	Ccnd1	Ccnd2	Ccne1	Ccne2	Cd44	Cdc16	Cdc23	Cdc27
		Cdc42	Cdk2	Cdk4	Chek1	Chek2	Chuk	Col1a1	Col1a2	Creb1	Creb3l2	Crebbp	Dvl1	Dvl3	E2f1	E2f2	E2f3
		Egfr	Eif2ak2	Eif2ak4	Eif2s1	Elk1	Ep300	Ets1	Fasl	Fn1	Foxo1	Fzd1	Fzd5	Fzd7	Gabarapl1	Grb2	Gsk3b
		Hdac2	Hdac3	Hif1a	Il6	Itga5	Itgav	Itgb1	Itgb2	Itgb3	Itpr1	Itpr3	Jun	Kat2b	Kras	Llgl1	Map2k7
		Map3k7	Mapk10	Mapk14	Mapk9	Mdm2	Mpp5	Mtor	Myc	Nfkb1	Nfkbia	Notch1	Notch2	Nras	Pard6a	Pdgfb	Pdgfrb
		Pdia3	Pik3cb	Pik3cd	Pik3r1	Pik3r2	Pik3r3	Ppp2ca	Ppp2cb	Ppp2r1b	Ppp2r2a	Prkaca	Prkacb	Prkca	Prkcb	Prkcg	Prkci
		Prkcz	Ptk2	Rac1	Rb1	Rbl1	Rbl2	Rela	Rps6kb1	Rxra	Skp2	Slc2a1	Smad2	Smad3	Smad4	Sos1	Sos2
		Src	Stat1	Stat3	Tcf7	Tcf7l1	Tcf7l2	Tgfb1	Tgfb2	Tgfb3	Tgfbr1	Tgfbr2	Tlr4	Usp7	Vegfa	Vtn	Wnt2
		Wnt5a	Ywhab	Ywhag	Ywhah	Zbtb17											
Proteoglycans in cancer	Group28	Actb	Actn4	Afdn	Akt1	Ank2	Ank3	Apc	Araf	Arhgef1	Arhgef12	Baiap2	Bcl2	Bcl2l11	Braf	Calm1	Calm2
		Calm3	Camk2a	Camk2d	Camk2g	Capn2	Casp3	Cblb	Ccnd1	Ccnd2	Ccne1	Ccne2	Cd44	Cdc42	Cdh1	Cdk2	Cdk4
		Chuk	Col1a1	Col1a2	Creb1	Creb3l2	Crk	Ctnnd1	Ctsl	Ddx5	Egfr	Eif4b	Elk1	Erbb2	Esr1	Ets1	Ezr
		Fasl	Fgfr1	Fgfr3	Flnb	Fn1	Foxo4	Frs2	Fyn	Fzd1	Fzd5	Fzd7	G6pc	Gab1	Gpc1	Grb2	Gsk3b
		Hbegf	Hgf	Hif1a	Id1	Igf1	Igf1r	Il6	Il7r	Insr	Iqgap1	Irs1	Itga5	Itgav	Itgb1	Itgb2	Itgb3
		Itpr1	Itpr3	Jun	Kdr	Kras	Map2k7	Map3k5	Map3k7	Mapk10	Mapk14	Mapk9	Mdm2	Met	Mras	Mtor	Myc
		Nfkb1	Nfkbia	Nlk	Nras	Pak1	Pak2	Pak4	Pard6a	Pck1	Pdgfb	Pdgfrb	Pik3cb	Pik3cd	Pik3r1	Pik3r2	Pik3r3
		Pikfyve	Pip4k2a	Pip4k2b	Pip4k2c	Pip5k1a	Pip5k1c	Plcb1	Plce1	Ppp1ca	Ppp1cb	Ppp1r12a	Ppp1r12b	Ppp1r12c	Ppp2ca	Ppp2cb	Ppp2r1b
		Ppp2r2a	Prkaa1	Prkaa2	Prkaca	Prkacb	Prkca	Prkcb	Prkcg	Prkci	Prkcz	Ptk2	Ptpn11	Rac1	Rac2	Rap1a	Rap1b
		Rbl2	Rdx	Rela	Rock1	Rock2	Rps6kb1	Rras	Rras2	Rxra	Sdc1	Sdc2	Sdc4	Smad2	Sos1	Sos2	Src
		Stat1	Stat3	Stk11	Stk3	Stk4	Tfap4	Tgfb1	Tgfb2	Tgfb3	Tgfbr1	Tgfbr2	Tiam1	Timp3	Tlr4	Vav2	Vcl
		Vegfa	Vtn	Wasf2	Wasl	Wnt2	Wnt5a	Ywhab	Ywhag	Ywhah							
Rap1 signaling pathway	Group29	Actb	Actn4	Afdn	Akt1	Araf	Arhgef12	Bcl2	Bmp7	Bmpr2	Braf	Calm1	Calm2	Calm3	Camk2a	Camk2d	Camk2g
		Capn2	Casp3	Ccnd1	Ccnd2	Cdc42	Cdh1	Chuk	Col1a1	Col1a2	Crk	Ctnnd1	Egfr	Elk1	Erbb2	Ezr	Fasl
		Fgfr1	Fgfr3	Flnb	Fn1	Fyn	Gab1	Grb2	Gsk3b	Hgf	Id1	Igf1	Igf1r	Il6	Inpp5d	Insr	Itga5
		Itgav	Itgb1	Itgb2	Itgb3	Jun	Kdr	Kras	Map3k5	Mapk10	Mapk14	Mapk9	Met	Mras	Nfkb1	Nfkbia	Nras
		Pak1	Pak2	Pak4	Pard6a	Pdgfb	Pdgfrb	Pdk1	Pik3cb	Pik3cd	Pik3r1	Pik3r2	Pik3r3	Pip5k1a	Pip5k1c	Plcb1	Plce1
		Ppp1ca	Ppp1cb	Ppp1r12a	Ppp1r12b	Ppp1r12c	Ppp2ca	Ppp2cb	Ppp2r1b	Ppp2r2a	Prkaca	Prkacb	Prkca	Prkcb	Prkcg	Prkci	Prkcz
		Ptk2	Ptpn11	Rac1	Rac2	Rap1a	Rap1b	Rela	Rock1	Rock2	Rps6kb1	Rras	S1pr1	S1pr4	Sos1	Sos2	Src
		Stat1	Stat3	Tgfb1	Tgfb2	Tgfb3	Tiam1	Tlr4	Vav2	Vcl	Vegfa	Vtn	Wasf2	Wasl	Wnt5a		
ErbB signaling pathway	Group30	Acsbg1	Acsl3	Acsl6	Akt1	Anapc1	Anapc10	Anapc2	Anapc4	Anapc7	Araf	Atg12	Atp1a1	Atp1a2	Atp1b1	Atp1b2	Atp1b3
		Bcl2	Bcl2l11	Braf	Bub1	Calm1	Calm2	Calm3	Camk2a	Camk2d	Camk2g	Casp3	Cblb	Ccna2	Ccnd1	Ccnd2	Ccne1
		Ccne2	Cdc16	Cdc23	Cdc25a	Cdc27	Cdc42	Cdk2	Cdk4	Chuk	Col1a1	Col1a2	Cpt1a	Creb1	Creb3l2	Crk	Ctsl
		Dvl1	Dvl3	Egfr	Ehmt1	Eif2ak4	Eif2s1	Eif4b	Elk1	Erbb2	Ern1	Esr1	Fasl	Fgfr1	Fgfr3	Fn1	Foxo1
		Frs2	Fzd1	Fzd5	Fzd7	G6pc	Gab1	Gabarapl1	Gck	Grb2	Gsk3b	Hbegf	Hdac2	Hgf	Hif1a	Hk1	Hk2
		Igf1	Igf1r	Il6	Il7r	Insr	Irs1	Itga5	Itgav	Itgb1	Itgb3	Itpr1	Jun	Kdr	Kras	Lef1	Map2k7
		Map3k5	Map3k7	Mapk10	Mapk14	Mapk9	Mdm2	Met	Mras	Mtor	Myc	Nfkb1	Nfkbia	Nras	Pak1	Pak2	Pak4
		Pck1	Pdgfb	Pdgfrb	Pfkp	Pik3cb	Pik3cd	Pik3r1	Pik3r2	Pik3r3	Ppp1ca	Ppp1cb	Ppp2ca	Ppp2cb	Ppp2r1b	Ppp2r2a	Prkaa1
		Prkaa2	Prkab2	Prkaca	Prkacb	Prkag2	Prkca	Prkcb	Prkcg	Prkci	Prkcz	Ptk2	Ptpn1	Ptpn11	Ptprf	Rac1	Rap1a
		Rap1b	Rbl2	Rela	Rps6kb1	Rras	Rras2	Rxra	Skp2	Slc2a1	Slc7a5	Sod2	Sos1	Sos2	Src	Stat1	Stat3
		Stk11	Tbc1d4	Tcf7	Tcf7l1	Tcf7l2	Tgfb1	Tlr4	Vegfa	Vtn	Wnt2	Wnt5a	Xbp1	Ywhab	Ywhag	Ywhah	
Renal cell carcinoma	Group31	Actb	Actn4	Afdn	Akt1	Araf	Arhgef1	Arhgef12	Arnt	Arnt2	Atm	Atp1a1	Atp1a2	Atp1b1	Atp1b2	Atp1b3	Atp2a2
		Atr	Bak1	Bcl2	Braf	Calm1	Calm2	Calm3	Calr	Camk2a	Camk2d	Camk2g	Capn2	Casp3	Cblb	Ccnd1	Ccnd2
		Cdc42	Cdh1	Cdk4	Chek1	Chuk	Col1a1	Col1a2	Creb1	Creb3l2	Crebbp	Crk	Ctnnd1	Cul1	Cul2	Dgka	Dgkd
		Dgke	Dgkh	E2f1	E2f2	E2f3	Egfr	Egln1	Egln2	Egln3	Elk1	Eloc	Ep300	Epas1	Erbb2	Ets1	Fasl
		Fgfr1	Fgfr3	Fh1	Flnb	Fn1	Foxo4	Frs2	Fyn	Gab1	Grb2	Gsk3b	Hbegf	Hgf	Hif1a	Id1	Igf1
		Igf1r	Il6	Insr	Irs1	Itga5	Itgav	Itgb1	Itgb2	Itgb3	Itpr1	Itpr3	Jun	Kdr	Kras	Map2k7	Map3k5
		Map3k7	Mapk10	Mapk14	Mapk9	Mdm2	Met	Mras	Mtor	Myc	Nfkb1	Nfkbia	Nlk	Nras	Pak1	Pak2	Pak4
		Pard6a	Pdgfb	Pdgfrb	Pdia3	Pik3cb	Pik3cd	Pik3r1	Pik3r2	Pik3r3	Pip5k1a	Pip5k1c	Plcb1	Plce1	Plk3	Ppp1ca	Ppp1cb
		Ppp1r12a	Ppp1r12b	Ppp1r12c	Prkaca	Prkacb	Prkca	Prkcb	Prkcg	Prkci	Prkcz	Ptk2	Ptpn11	Rac1	Rac2	Rap1a	Rap1b
		Rb1	Rela	Rock1	Rock2	Rps6kb1	Rras	Rras2	Slc2a1	Sos1	Sos2	Src	Stat1	Stat3	Stk3	Stk4	Tgfb1
		Tgfb2	Tgfb3	Tgfbr1	Tgfbr2	Tiam1	Tlr4	Vav2	Vcl	Vegfa	Vhl	Vtn	Wasf2	Wasl	Wee1		
Hepatocellular carcinoma	Group32	Actb	Akt1	Apc	Araf	Atp1a1	Atp1a2	Atp1b1	Atp1b2	Atp1b3	Axin1	Bak1	Bcl2	Bmp4	Bmpr1a	Bmpr2	Braf
		Calm1	Calm2	Calm3	Camk2a	Camk2d	Camk2g	Casp3	Cblb	Ccna2	Ccnd1	Ccne1	Ccne2	Cdh1	Cdk2	Cdk4	Chuk
		Cpt1a	Creb1	Creb3l2	Crk	Dgka	Dgkd	Dgke	Dgkh	Dvl1	Dvl3	E2f1	E2f2	E2f3	Egfr	Ehmt1	Eif4b
		Elk1	Erbb2	Esr1	Fasl	Fgfr1	Fgfr3	Foxo1	Fyn	Fzd1	Fzd5	Fzd7	G6pc	Gab1	Gck	Grb2	Gsk3b
		Hbegf	Hdac2	Hgf	Hif1a	Hk1	Hk2	Id1	Id2	Igf1	Igf1r	Inpp5d	Insr	Irs1	Itgb2	Jun	Kras
		Lef1	Map2k7	Map3k7	Mapk10	Mapk14	Mapk9	Mdm2	Met	Mtor	Myc	Ncoa1	Ncoa3	Nfkb1	Nfkbia	Notch1	Notch2
		Nras	Pak1	Pak2	Pak4	Pck1	Pdgfb	Pdgfrb	Pfkp	Pik3cb	Pik3cd	Pik3r1	Pik3r2	Pik3r3	Pip5k1a	Pip5k1c	Ppp1ca
		Ppp1cb	Ppp2ca	Ppp2cb	Ppp2r1b	Ppp2r2a	Prkaa1	Prkaa2	Prkab2	Prkaca	Prkacb	Prkag2	Prkca	Prkcb	Prkcg	Prkci	Prkcz
		Ptk2	Ptpn1	Ptpn11	Ptprf	Rac1	Rac2	Rb1	Rela	Rps6kb1	Rxra	Setdb1	Skp2	Slc7a5	Smad1	Smad2	Smad3
		Smad4	Sod2	Sos1	Sos2	Src	Stat3	Stk11	Stk4	Tcf7	Tcf7l1	Tcf7l2	Tgfb1	Tgfb2	Tgfb3	Tgfbr1	Tgfbr2
		Vav2	Wasf2	Wasl	Wnt2	Wnt5a											
Human cytomegalovirus infection	Group33	Actb	Afdn	Akt1	Anapc1	Anapc10	Anapc2	Anapc4	Anapc7	Apc	Ar	Araf	Arhgef1	Arhgef12	Arnt	Atp1a1	Atp1a2
		Atp1b1	Atp1b2	Atp1b3	Atp2a2	Axin1	Bak1	Bcl2	Braf	Bub1	Calm1	Calm2	Calm3	Calr	Camk2a	Camk2d	Camk2g
		Casp3	Cblb	Ccn2	Ccnd1	Ccnd2	Ccne1	Ccne2	Cdc16	Cdc23	Cdc27	Cdc42	Cdh1	Cdk2	Cdk4	Chuk	Col1a1
		Col1a2	Cpt1a	Creb1	Creb3l2	Crebbp	Crk	Csnk1e	Csnk2a1	Cul1	Cyp4a10	Cyp4a12a	Dvl1	Dvl3	E2f1	E2f2	E2f3
		Egfr	Elk1	Ep300	Erbb2	Esr1	Ezr	Fasl	Fh1	Foxo1	Frs2	Fyn	Fzd1	Fzd5	Fzd7	G6pc	Gab1
		Gabarapl1	Gck	Grb2	Gsk3b	Hbegf	Igf1	Igf1r	Il6	Inpp5d	Irs1	Itgav	Itgb1	Itgb3	Itpr1	Itpr3	Jun
		Klf2	Kras	Ldha	Lef1	Map2k7	Map3k5	Map3k7	Mapk10	Mapk14	Mapk9	Mdm2	Mras	Mtor	Myc	Ncoa1	Ncoa2
		Ncoa3	Nfkb1	Nfkbia	Nlk	Nras	Pak1	Pak2	Pak4	Pck1	Pdgfb	Pdgfrb	Pdha1	Pdhb	Pdia3	Pgam1	Pik3cb
		Pik3cd	Pik3r1	Pik3r2	Pik3r3	Plcb1	Plce1	Plk3	Ppp1ca	Ppp1cb	Ppp1r12a	Ppp1r12b	Ppp1r12c	Ppp2ca	Ppp2cb	Ppp2r1b	Ppp2r2a
		Prkaa1	Prkaa2	Prkab2	Prkaca	Prkacb	Prkag2	Prkca	Prkcb	Prkcg	Prkci	Prkcz	Prmt1	Ptk2	Ptpn11	Rac1	Rac2
		Rap1a	Rap1b	Rb1	Rela	Rock1	Rock2	Rps6kb1	Rras	Rras2	Serpine1	Slc2a1	Smad2	Smad3	Smad4	Smc1a	Smc3
		Sos1	Sos2	Src	Stat1	Stat3	Tcf7	Tcf7l1	Tcf7l2	Tgfb1	Tgfbr1	Tgfbr2	Tiam1	Vav2	Vegfa	Wnt2	Wnt5a
		Ywhab	Ywhag	Ywhah													
Cell cycle	Group34	Actb	Actn4	Afdn	Akt1	Anapc1	Anapc10	Anapc2	Anapc4	Anapc7	Apc	Ar	Araf	Arhgef1	Arhgef12	Arnt	Arnt2
		Atg12	Atm	Atp1a1	Atp1a2	Atp1b1	Atp1b2	Atp1b3	Atp2a2	Atr	Axin1	Bak1	Bbc3	Bcl2	Bcl2l11	Bmp4	Bmp7
		Bmpr1a	Bmpr2	Braf	Bub1	Bub3	Calm1	Calm2	Calm3	Calr	Camk2a	Camk2d	Camk2g	Canx	Capn2	Casp3	Cblb
		Ccna2	Ccnd1	Ccnd2	Ccne1	Ccne2	Cd44	Cdc14a	Cdc14b	Cdc16	Cdc23	Cdc25a	Cdc27	Cdc42	Cdc7	Cdh1	Cdk2
		Cdk4	Cdk7	Chek1	Chek2	Chuk	Col1a1	Col1a2	Creb1	Creb3l2	Crebbp	Crk	Csnk2a1	Ctnnd1	Ctsl	Cul1	Cul2
		Cyp4a10	Cyp4a12a	Dgka	Dgkd	Dgke	Dgkh	Dvl1	Dvl3	E2f1	E2f2	E2f3	E2f5	Egfr	Egln1	Egln2	Egln3
		Ehmt1	Eif2ak2	Eif2ak4	Eif2s1	Eif4b	Elk1	Eloc	Ep300	Epas1	Erbb2	Ern1	Esr1	Ets1	Ezr	Fasl	Fgfr1
		Fgfr3	Fh1	Flnb	Fn1	Foxo1	Foxo4	Frs2	Fyn	Fzd1	Fzd5	Fzd7	G6pc	Gab1	Gabarapl1	Gck	Gpc1
		Grb2	Gsk3b	Hbegf	Hdac2	Hdac3	Hgf	Hif1a	Hk1	Hk2	Id1	Id2	Igf1	Igf1r	Il6	Il7r	Inpp5d
		Insr	Irs1	Itga5	Itgav	Itgb1	Itgb2	Itgb3	Itpr1	Itpr3	Jun	Kat2b	Kdr	Klf2	Kras	Lef1	Llgl1
		Map2k7	Map3k5	Map3k7	Mapk10	Mapk14	Mapk9	Mcm3	Mcm4	Mcm5	Mcm7	Mdm2	Met	Mpp5	Mras	Mtor	Myc
		Ncoa1	Ncoa2	Ncoa3	Nfkb1	Nfkbia	Nlk	Notch1	Notch2	Nras	Orc2	Orc4	Pak1	Pak2	Pak4	Pard6a	Pck1
		Pdgfb	Pdgfrb	Pdia3	Pdk1	Pik3cb	Pik3cd	Pik3r1	Pik3r2	Pik3r3	Pip5k1a	Pip5k1c	Plcb1	Plcd1	Plce1	Plk3	Ppp1ca
		Ppp1cb	Ppp1r12a	Ppp1r12b	Ppp1r12c	Ppp2ca	Ppp2cb	Ppp2r1b	Ppp2r2a	Prkaa1	Prkaa2	Prkab2	Prkaca	Prkacb	Prkag2	Prkca	Prkcb
		Prkcg	Prkci	Prkcz	Prkdc	Ptk2	Ptpn1	Ptpn11	Ptprf	Rac1	Rac2	Rad21	Rap1a	Rap1b	Rb1	Rbl1	Rbl2
		Rela	Ret	Rock1	Rock2	Rps6kb1	Rras	Rras2	Rxra	S1pr1	S1pr4	Sdc1	Sdc2	Sdc4	Serpine1	Setdb1	Skp2
		Slc2a1	Slc7a5	Smad1	Smad2	Smad3	Smad4	Smc1a	Smc3	Sod2	Sos1	Sos2	Src	Stag1	Stag2	Stat1	Stat3
		Stk11	Stk3	Stk4	Tcf7	Tcf7l1	Tcf7l2	Tfdp1	Tfdp2	Tgfb1	Tgfb2	Tgfb3	Tgfbr1	Tgfbr2	Tiam1	Tlr4	Usp7
		Vav2	Vcl	Vegfa	Vhl	Vtn	Wasf2	Wasl	Wee1	Wnt2	Wnt5a	Xbp1	Ywhab	Ywhag	Ywhah	Zbtb17	
