# Supplementary material for: Adipose tissue from metabolic syndrome mice induces an aberrant miRNA signature highly relevant in prostate cancer development
Source: Mol Oncol. 2020 Sep 25;14(11):2868–83. doi: 10.1002/1878-0261.12788 (PMC7607170; doi:10.1002/1878-0261.12788)
Supplement: Supplementary file 9 — Table S9. Functional enrichment of target genes present only in up modulated miRNAs. [file MOL2-14-2868-s009.pdf]

Table S9. Functional enrichment of target genes present only in up modulated miRNAs. ClueGo

Function	Groups	Group Genes												
Purine metabolism	Group00	Hprt	Pgm1	Ppat	Prps1	Rrm1	Rrm2b	Uox	Xdh					
Caffeine metabolism	Group01	Cyp1a2	Uox	Xdh										
Steroid biosynthesis	Group02	Cyp51	Dhcr24	Ebp	Msmo1	Sc5d	Sqle							
Primary bile acid biosynthesis	Group03	Baat	Cyp27a1	Hsd3b7										
Oxidative phosphorylation	Group04	Atp5a1	Atp5f1	Atp5o	Atp6v1d	Cox8a	Ndufa13	Uqcrc1	Uqcrfs1					
Cysteine and methionine metabolism	Group05	Adi1	Gclc	Gclm	Mat1a	Mat2a	Mri1	Sds	Tat					
Glutathione metabolism	Group06	Gclc	Gclm	Ggt6	Rrm1	Rrm2b								
Glycerolipid metabolism	Group07	Akr1a1	Aldh1b1	Aldh9a1	Dgat2	Gpam	Lpin2	Mgll	Tkfc					
Alanine, aspartate and glutamate metabolism	Group08	Cps1	Gfpt1	Glul	Otc	Ppat								
Pyruvate metabolism	Group09	Aass	Aldh1b1	Aldh9a1	Grhpr	Me1								
Glyoxylate and dicarboxylate metabolism	Group10	Alas2	Cs	Gldc	Glul	Grhpr	Hao1	Sds						
Pentose phosphate pathway	Group11	Akr1a1	Aldh1b1	Aldh9a1	Aldob	Fbp1	Gpi1	H6pd	Pfkl	Pgm1	Prps1	Rgn	Sord	Tkfc
Ascorbate and aldarate metabolism	Group12	Akr1a1	Alas2	Aldh1b1	Aldh9a1	Ces1d	Cyp1a2	Cyp2a5	Cyp2c70	Cyp3a11	Dpyd	Fech	Hprt	Rdh11
		Rgn	Rrm1	Rrm2b	Sord	Ugt1a1	Ugt1a5	Ugt1a7c	Ugt2b34	Ugt2b36	Ugt2b5	Xdh		
